# *Tfap2a*-dependent changes in mouse facial morphology result in clefting that can be ameliorated by a reduction in *Fgf8* gene dosage

**DOI:** 10.1242/dmm.017616

**Published:** 2014-11-07

**Authors:** Rebecca M. Green, Weiguo Feng, Tzulip Phang, Jennifer L. Fish, Hong Li, Richard A. Spritz, Ralph S. Marcucio, Joan Hooper, Heather Jamniczky, Benedikt Hallgrímsson, Trevor Williams

**Affiliations:** 1 Department of Craniofacial Biology, University of Colorado Denver, 12801 East 17th Avenue, Aurora, CO 80045, USA.; 2 Department of Pharmacology, University of Colorado Denver, 12801 East 17th Avenue, Aurora, CO 80045, USA.; 3 University of California San Francisco, Orthopaedic Trauma Institute, Department of Orthopaedic Surgery, San Francisco, CA 94110, USA.; 4 Human Medical Genetics and Genomics Program, University of Colorado School of Medicine, 12800 East 17th Avenue, Aurora, CO 80045, USA.; 5 Department of Cell and Developmental Biology, University of Colorado Denver, 12801 East 17th Avenue, Aurora, CO 80045, USA.; 6 McCaig Institute for Bone and Joint Health, Department of Cell Biology & Anatomy, University of Calgary, 3280 Hospital Drive NW, Calgary, AB T2N3Z6, Canada.; 7 Alberta Children’s Hospital Research Institute, University of Calgary, 3280 Hospital Drive NW, Calgary, AB T2N3Z6, Canada.

**Keywords:** Craniofacial, *TFAP2A*, AP-2α, BOFS, Branchio-oculofacial syndrome, Cleft lip/palate, Geometric morphometrics, Fgf signaling pathway

## Abstract

Failure of facial prominence fusion causes cleft lip and palate (CL/P), a common human birth defect. Several potential mechanisms can be envisioned that would result in CL/P, including failure of prominence growth and/or alignment as well as a failure of fusion of the juxtaposed epithelial seams. Here, using geometric morphometrics, we analyzed facial outgrowth and shape change over time in a novel mouse model exhibiting fully penetrant bilateral CL/P. This robust model is based upon mutations in *Tfap2a*, the gene encoding transcription factor AP-2α, which has been implicated in both syndromic and non-syndromic human CL/P. Our findings indicate that aberrant morphology and subsequent misalignment of the facial prominences underlies the inability of the mutant prominences to fuse. Exencephaly also occured in some of the *Tfap2a* mutants and we observed additional morphometric differences that indicate an influence of neural tube closure defects on facial shape. Molecular analysis of the CL/P model indicates that Fgf signaling is misregulated in the face, and that reducing *Fgf8* gene dosage can attenuate the clefting pathology by generating compensatory changes. Furthermore, mutations in either *Tfap2a* or *Fgf8* increase variance in facial shape, but the combination of these mutations restores variance to normal levels. The alterations in variance provide a potential mechanistic link between clefting and the evolution and diversity of facial morphology. Overall, our findings suggest that CL/P can result from small gene-expression changes that alter the shape of the facial prominences and uncouple their coordinated morphogenesis, which is necessary for normal fusion.

## INTRODUCTION

Orofacial clefts are common human birth defects, with cleft lip with or without cleft palate (CL/P) occurring in about one in 700 births, and cleft palate only (CP) presenting about half as frequently (Mossey and Modell, 2012). Although many instances of orofacial clefts are syndromic and associated with a specific developmental syndrome, the majority of clefts are isolated and non-syndromic (Mossey and Modell, 2012; Rahimov et al., 2012).

Face shape has long been considered a factor in the development of CL/P. Studies of unaffected parents of children with CL/P using cephalometry, or more recently detailed three-dimensional (3D) morphometrics, show distinct differences in facial shape compared with unaffected families (McIntyre and Mossey, 2002; Nakasima and Ichinose, 1983; Wyszynski, 2002; Weinberg et al., 2006; Weinberg et al., 2009). These differences hold true for many different racial and ethnic backgrounds, and often include a more concave face.

In mouse, genetic manipulations tend to result in CP, rather than CL/P (which is more common in humans) (Juriloff and Harris, 2008). This difference presumably reflects a combination of alterations in facial shape, growth, morphogenesis and gene expression between these species. The greater prevalence of mutations leading to CP in mouse, combined with the relative ease of culturing isolated palatal shelves, has generated a better understanding of the mechanisms involved in the formation of the secondary palate. CP can result from a number of specific causes, including defects in growth and morphogenesis, defective fusion following apposition, mechanical defects caused by aberrant positioning of the tongue, or inappropriate fusion of the palatal epithelia to other oral epithelia (Bush and Jiang, 2012; Gritli-Linde, 2007). Studies on CL/P have not advanced as far, in part because, until recently, there were few models that displayed CL/P with high penetrance.

In both human and mouse, facial development begins with the appearance of discrete buds of tissue, termed the facial prominences (Brugmann et al., 2006; Chai and Maxson, 2006). Three pairs of prominences form the region of the upper jaw: the medial nasal, the lateral nasal and the maxillary. In mouse development, at embryonic day (E)9.5 the prominences have begun to form and, by E10.5, they are undergoing rapid outgrowth. The medial nasal prominences have met at the midline by E11.5, and the other prominences are in close apposition and are beginning to fuse. By E12.5 they have fused to create a continuous tissue band at the front of the face – the primary palate (Brugmann et al., 2006; Chai and Maxson, 2006; Ray and Niswander, 2012). Although the basic steps required for fusion are known, it is unclear which step or steps fail during the development of CL/P. There are two major and non-exclusive hypotheses for the etiology of CL/P: the first is that the epithelium at these locations is somehow incapable of fusing, whereas the second is that the shape of the face and the direction of growth of the facial prominences are altered such that these structures are not aligned to allow for normal fusion (Ferretti et al., 2011; Trasler, 1968).

**TRANSLATIONAL IMPACT**

**Clinical issue**

Orofacial clefting is one of the most frequent human birth defects, with cleft lip with or without cleft palate (CL/P) affecting between 1 in 500 to 1 in 1000 live births worldwide. Clefting of the secondary palate (CP; the roof of the mouth) occurs slightly less frequently. Clefting is treatable in humans, but even with treatment this condition can have serious consequences for speech, feeding and social interactions. CL/P or CP treatment requires a complex and multilayered approach, which includes successive surgeries, speech therapy and orthodontics; lifelong medical treatment is required for many individuals. Whereas, in humans, CL/P is more common than CP, most mouse models display CP. In addition, the classic mouse models of CL/P were hampered by partial penetrance of the phenotype.

**Results**

This study describes the generation of a new and a fully penetrant mouse model of CL/P caused by mutations in *Tfap2a*, a gene linked to human CL/P. With this model it is possible to study the gene expression changes underlying CL/P as well as to examine whether clefting results from altered facial shape or altered fusion events. The methodology of geometric morphometrics was employed to compare how facial shape changes between control and mutant embryos affects facial morphology. In the mutant model, clefting develops due to altered shape of the upper facial prominences, which changes prominence alignment, preventing fusion of the primary palate. Global gene expression analysis shows that alterations in gene expression in mutant mice are relatively minor despite the severity of the resulting pathology. However, alterations in the fibroblast growth factor (Fgf) signaling pathway are evident and decreasing the gene dosage of Fgf8 can generate a partial rescue of the phenotype.

**Implications and future directions**

This work suggests that the formation of the lip and primary palate is particularly sensitive to small changes in growth and gene expression. This finding might explain why this developmental process often goes awry in human development owing to genetic and/or environmental causes. Moreover, the finding that the clefting pathology in this model can be altered by manipulation of Fgf signaling during embryonic facial development could one day lead to directed early pharmacological interventions, rectifying facial growth and preventing the need for repetitive surgeries.

Geometric morphometrics provides a powerful means to evaluate the role of shape in the development of fusion defects of the upper lip and primary palate (Cooper and Albertson, 2008; Martínez-Abadías et al., 2012). Geometric morphometrics uses homologous-points-based data (landmarks) that are then scaled and superimposed for shape analysis by multivariate statistical methods (Klingenberg, 2010). In mouse, geometric morphometrics has been used to probe how facial shape might influence orofacial clefting in two strains that are prone to CL/P, A/WySn and CL/Fr (Parsons et al., 2008; Young et al., 2007). However, these two strains have a relatively low penetrance of CL/P so that it is only possible to determine overall trends in shape difference because embryos that will go on to form a cleft cannot be distinguished from embryos that will develop normally. Nevertheless, differences in growth and patterning are likely involved, because these influence the morphogenesis of 3D shape (Boehm et al., 2010; Martínez-Abadías et al., 2013). Indeed, Fgf, Hh, Bmp and Wnt signaling molecules are important for patterning the midface, and decreased Fgf signaling has been linked to CL/P in humans (Carroll et al., 2005; Griffin et al., 2013; Hu and Marcucio, 2009; Jin et al., 2012; Riley et al., 2007). However, it is currently unclear how these pathways are integrated to control facial development.

Here, we describe a new mouse model of CL/P caused by mutations in *Tfap2a*, the gene encoding transcription factor AP-2α. Mutant mice display fully penetrant bilateral orofacial clefting, and provide an excellent means to analyze how this pathology progresses with respect to a defect in either alignment or epithelial fusion. Specifically, we can employ geometric morphometrics to test the hypothesis that there are alterations in facial morphology that correlate with the development of orofacial clefting. In humans, *TFAP2A* is mutated in branchio-oculo-facial syndrome (BOFS) and regulates, or is regulated by, additional genes associated with orofacial clefting, including *Irf6*, *BCOR* and *p63* (Fan et al., 2009; Ferretti et al., 2011; Gritli-Linde, 2010; Li et al., 2013a; Milunsky et al., 2008; Rahimov et al., 2008; Stoetzel et al., 2009; Thomason et al., 2010; Wang et al., 2013). Therefore, our findings are directly relevant to the genetic underpinnings of human CL/P and provide mechanistic insight into how such pathology can be modified.

## RESULTS

### Derivation of the Neo/Null model of bilateral facial clefting

**Fig. 1.**
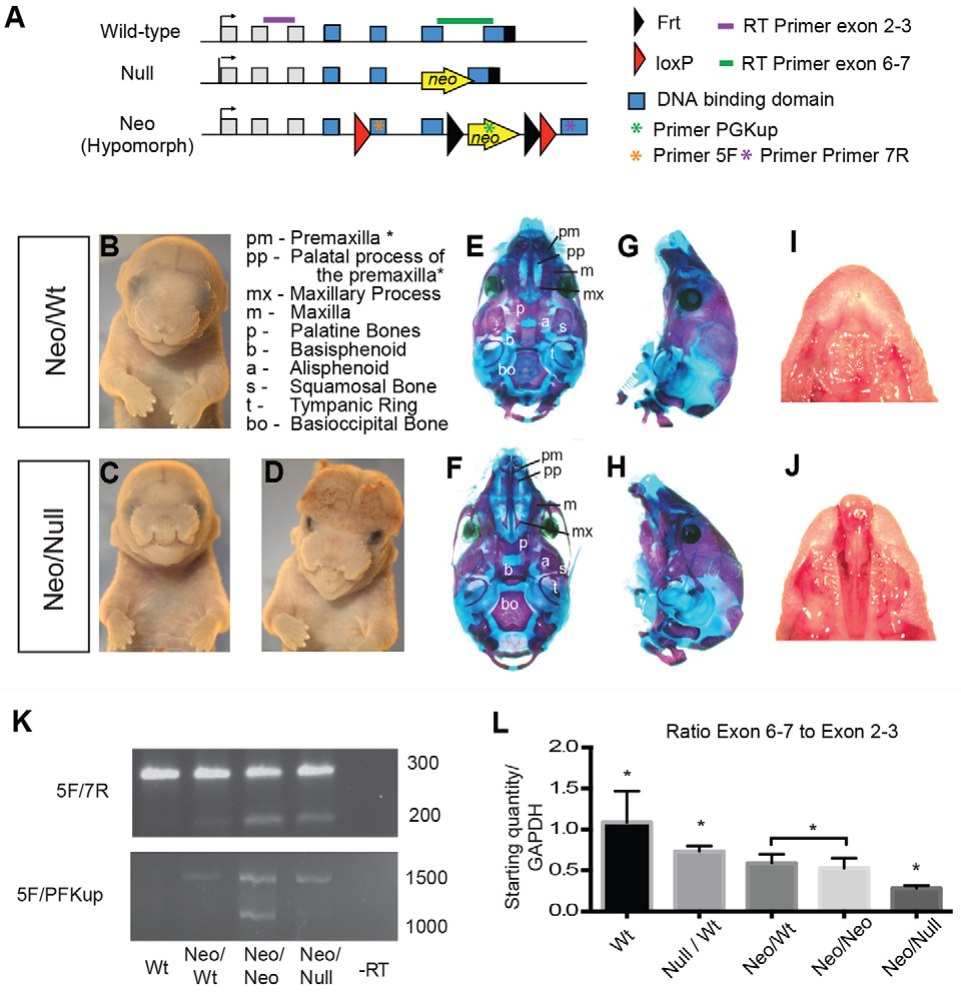
**The Neo/Null allele and the bilateral cleft phenotype.** (A) Schematic diagram of the *Tfap2a* alleles. The Neo/Null model is a combination of the Neo (Brewer et al., 2004) and the *Tfap2a* null (Zhang et al., 1996) alleles. The positions of primers for RT-PCR and qRT-PCR are shown, along with the intron:exon structure of the gene, and the position of sequences introduced by gene targeting. See Materials and Methods for details of primers. Frt, flippase recombination target. (B–D) E18.5 embryos showing the control phenotype (B), and the bilateral cleft phenotype in Neo/Null with normal neural tube closure (C) and Neo/Null with exencephaly (D). (E–H) Bone and cartilage staining from P1 Neo/Wt (E,G) and Neo/Null (F,H) after mandible removal showing norma basilaris (E,F) and norma lateralis (G,H). An asterisk after the name of the bone represents an area where differences were observed between Neo/Wt control and Neo/Null embryos. (I,J) Images of the palate from Neo/Wt (I) and Neo/Null (J) E18.5 embryos. Note the cleft in J extending from the primary palate into the secondary. (K) RT-PCR analysis of the *Tfap2a* transcripts that are present in the E10.5 face using the primer pairs shown in A. ‘-RT’ is a no reverse transcriptase control, and ‘Wt’ is wild type. (L) qRT-PCR data showing the ratio of *Tfap2a* transcripts containing exons 6–7 to those containing exons 2–3. Transcripts lacking exon 6–7 would lack the DNA-binding domain. *P<0.05 to all other genotypes. The two genotypes marked by a bar are not statistically different from each other, but they are different from all other alleles.

Loss of *Tfap2a* in the mouse results in major defects in development of the head, with widely separated mandibular and maxillary processes as well as exencephaly (Schorle et al., 1996; Zhang et al., 1996). More recently, several conditional *Tfap2a* alleles have been generated (Brewer et al., 2004), including the Neo allele (Fig. 1A), in which the *neomycin resistance* (*neo*) cassette is present between exons encoding part of the AP-2α DNA-binding domain (Williams and Tjian, 1991a; Williams and Tjian, 1991b). Because the *neo* insertion can create a hypomorphic allele in other instances (Hester et al., 2005; Meyers et al., 1998), we compared the phenotypes of mice that were heterozygous or homozygous for the Neo allele, as well as mice that were transheterozygous for the Neo and null alleles (Neo/Null). In this *Tfap2a* allelic series, Neo homozygotes and heterozygotes were normal and viable, whereas E18.5 Neo/Null mice had fully penetrant bilateral facial clefting with concomitant perinatal lethality (Fig. 1B,C). For further study, homozygous Neo mice were bred to heterozygous null (*Tfap2a*^+/−^) mice to generate 50% affected Neo/Null mice and 50% Neo*/*Wt mice. The latter had a normal appearance and served as the control group. Neo/Null embryos also showed additional gross external abnormalities, including polydactyly and mid/hindbrain exencephaly but at a lower frequency than CL/P (Fig. 1D and data not shown). With relevance for head formation, exencephaly occurred in ~30% of the E18.5 Neo/Null mice, compared with about a 12% incidence of neural tube closure defects in *Tfap2a^+/−^* mice (Kohlbecker et al., 2002) (R.M.G. and T.W., data not shown). Gross brain morphology in Neo/Wt and Neo/Null mice with normal neural tube closure was comparable, indicating that the mutants did not display a general disruption of brain patterning (data not shown). Although 30% of Neo/Null animals had exencephaly at E18.5, at E9.5 ~45% had abnormal neural tube closure, indicating that some embryos have a delay in this process that eventually resolves. Because more than 50% of Neo/Null embryos had normal neural tube closure at E9.5 but still developed bilateral CL/P, the CL/P phenotype could be studied either in isolation or in conjunction with the exencephaly.

Bone and cartilage staining of controls showed that the premaxilla formed a continuous arc from the midline to the maxilla, whereas, in the Neo/Null embryos (with closed neural tubes), the premaxilla was discontinuous (Fig. 1E–H). This resulted in the premaxilla protruding from the anterior of the face as a bulbous structure. Although both the primary and secondary palates were affected in the Neo/Null mice (Fig. 1I,J), the secondary-palate defects were mainly associated with defects in the premaxilla and the premaxillary palatal processes. Thus, defects were generally not observed in the more posterior regions of the secondary palate, with the exception of a low penetrance of defects in the maxillary palatal processes (2/10 embryos).

Because Neo/Null mice are more severely affected than *Tfap2a^+/−^* mice, we next used reverse transcription (RT)-PCR to determine whether the *neo* insertion significantly altered the expression and/or processing of the associated transcripts from the Neo allele. RNA was isolated at E10.5 from the faces of wild-type controls, *Tfap2a*^+/−^ and Neo/Null mice, as well as Neo homozygotes and heterozygotes. Primer pairs located within exons 5 and 7 (Fig. 1A) detected a wild-type spliced product in all samples (Fig. 1K). However, they also revealed a shorter product in all mice carrying the Neo allele, and further analysis using primers within the *neo* cassette indicated that aberrantly spliced *Tfap2a* products were present in such mice (Fig. 1K). Next, we used quantitative RT-PCR to assess relative wild-type *Tfap2a* transcript levels. Primer pairs located in upstream exons (exons 2–3) of the *Tfap2a* locus indicated that this portion of the transcript was decreased nearly equally across all mice containing various mutant allele combinations, compared with wild type (supplementary material Fig. S1A). In contrast, primer pairs that detected only transcripts that were normally spliced between exons 6 through 7 showed that homozygous Neo mice contained only 35% of wild-type *Tfap2a* levels, and that Neo/Null mice contained less than 20% (supplementary material Fig. S1B). The ratio between exons 2–3 and exons 6–7 decreased successively for the Null heterozygote, mice with various *neo* allele combinations, and Neo/Null mice (Fig. 1L). Sequencing of RT-PCR products identified several splice variants (supplementary material Fig. S1C,D). One variant skips exon 6, whereas another includes this exon but then reads into the adjoining intron, and a third splices from exon 6 into the *neo* cassette. The variants would generate protein truncations and frameshifts that would abolish AP-2α DNA-binding and dimerization. These findings demonstrate that the Neo allele generates aberrant transcripts that would not encode functional AP-2α, as well as reducing the quantities of wild-type product. In conjunction with the phenotypes observed from previous AP-2α studies as well as in the allelic series, these findings suggest that, in the Neo/Null mice, functional *Tfap2a* levels drop below a critical threshold required for normal facial fusion.

### Independent shape differences associated with exencephaly and facial clefting

The Neo/Null model exhibits completely penetrant bilateral CL/P and provides a means to study the development of this pathology using geometric morphometrics during the critical period of normal facial fusion, between E9.5-E11.5. Because a significant fraction of the Neo/Null mice had exencephaly, we could also investigate how this pathology impacted facial shape. Thus, three groups were studied: Neo/Wt; Neo/Null with closed neural tube; and Neo/Null with open neural tube. As an initial analysis, a total of ~100 embryos between E9.5 and 11.5 were subjected to μCT scanning to generate detailed surface images. These surface images were then landmarked and analyzed using geometric morphometric methods. All data were regressed against tail somite number and centroid size to remove small differences within the age groups.

**Fig. 2.**
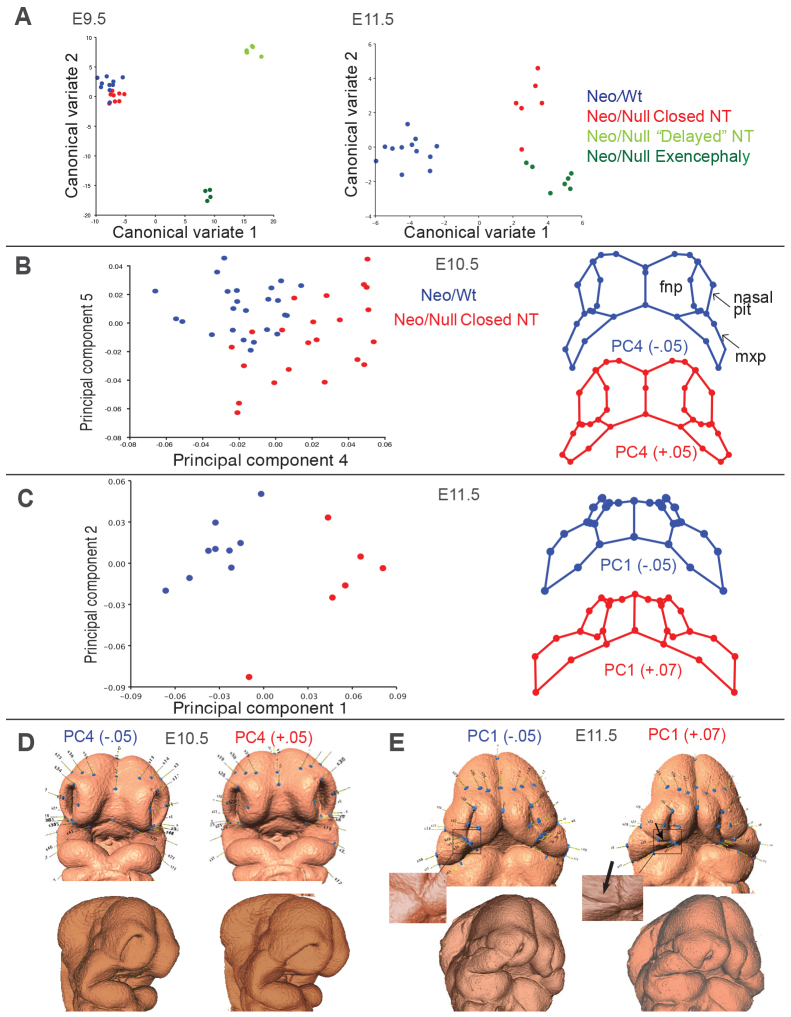
**Morphometric analysis of the orofacial clefting etiology.** (A) CVA of shape change at E9.5 and E11.5. Neural tube was categorized ‘closed’, ‘delayed’ or ‘exencephalic’ as shown in supplementary material Fig. S2. (B) Left: PCA of E10.5 embryos (23–35 somites from forelimb to tail tip) showing separation along PC4. Right: wireframes for this component show changes in the nasal pit and maxilla (mxp). fnp, nasal prominences. The wireframes represent a ‘typical’ embryo for that genotype and are based on the number shown under the wireframe (blue: Neo/Wt; red: Neo/Null). (C) Left: PCA of E11.5 (40–50 somite) embryos with separation along PC1. Right: wireframe changes are seen primarily in the size and shape of the nasal pit and the maxilla. (D) Application of the wireframes in B to a control E10.5 embryo. (E) Application of the wireframes in C to a control E11.5 embryo. The arrow denotes a region of high deformation under the nasal pit where the cleft will develop to the ‘Neo/Null’ side of PC1. The insets show this region in greater detail. Blue dots/lines show the landmarks used in the analysis.

E9.5 was the earliest time point investigated, because landmarking requires sufficient surface detail to mark consistent morphological features. At E9.5, 20 landmarks could be assigned and these could be correlated across all samples of all ages (Materials and Methods, and supplementary material Table S1). When scanning later time points, E10.5 and E11.5, there was sufficient surface detail to assign additional landmarks to the developing face. To compare groups and examine the between-group differences, a Procrustes permutation test was used, followed by a canonical variates analysis (CVA) to interpret the nature of the differences. At E9.5, there were statistically significant differences between all groups (Fig. 2A; supplementary material Table S2). Three groupings had large Procrustes distances, corresponding to embryos with cranial neural tubes that were closed, delayed or everted/presumptive exencephalic (supplementary material Fig. S2A shows representative raw scans of each grouping). In contrast, the two groups with a closed neural tube clustered more tightly together, despite their different genotypes. Therefore, at this time point, the most significant differences in facial shape correspond to the state of neural tube closure, rather than the *Tfap2a* genotype. Notably, embryos with an open neural tube had a narrower telencephalon that is likely to impact the associated frontonasal tissue. By E11.5, the groups separated more strongly by genotype, in addition to neural tube closure, creating three statistically distinct groups: Neo/Wt; Neo/Null open neural tube; and Neo/Null closed neural tube (Fig. 2A). The separation between the two Neo/Null groups indicates that the failure of neural tube closure independently affects facial shape.

### Facial clefting correlates with altered morphology of the nasal pit and maxilla

Next, we focused on the development of Neo/Wt (control) and Neo/Null (CL/P) embryos with appropriate neural tube closure to address specifically the shape changes that were involved in the development of the orofacial cleft, especially in the area around the newly forming nasal pit. The earliest time point examined was ~E10.25, equivalent to 23 or greater somites (Som) from tail tip to the forelimb, when the nasal pits first become visible and can be landmarked. At this stage, an established set of 48 landmarks that focus on the developing facial prominences and nasal pit could be used. The data were then analyzed using a principal component analysis (PCA) to determine the principal axes of variation. At this early developmental stage, 23–35 Som, the first three principal components (PC1–3) did not distinguish between the different genotypes (supplementary material Fig. S3). However, examination of PC4 indicates that alterations in facial morphology specific to the Neo/Null mutation can already be detected (Fig. 2B). PC4 accounts for ~8% of the overall shape variance (supplementary material Fig. S3). The morphological significance of PC4 can be observed through the deformation of the wireframe diagram at various points along the PC. The Neo/Wt embryos are clustered along the negative end and the Neo/Null along the positive. The values −0.05 and +0.05 were chosen to represent Neo/Wt and Neo/Null, respectively. Based on the wireframe deformations, at this stage, genotype correlated with changes in the shape of the nasal pits and maxilla (Fig. 2B,D). Because PC4 (genotype) accounts for only a small percentage of the overall morphological variation, the remaining variation might be due to rapid and major shape changes occurring during this period of facial development that are not fully removed by regressing on somite stage. In addition, some variation might be attributable to genetic background from the use of an outbred mouse strain (Black Swiss). In addition to PCA, we used CVA to visualize differences between the E10.25 Neo/Null and Neo/Wt embryos. The shape differences between embryos of these two genotypes at this stage, although relatively small, were highly significant (*P*<0.005; Procrustes permutation test; data not shown).

By E11.5 (40–50 Som), the percentage of the shape change that can be attributed to genotype increased, and was now seen in the first PC, which accounted for about 30% of the total shape variance. Based on the wireframe deformation graph for PC1, drastic changes could be seen in both the relative size and shape of the nasal pit and the maxilla (Fig. 2C). Furthermore, it seems that, when a typical control embryo is warped to fit the landmark coordinates that would resemble a typical Neo/Null embryo, a notch begins to form underneath the nasal pit in the area where a cleft will form (Fig. 2E, arrow), suggesting that a quantitative deformation of the control facial shape to match the Neo/Null points is sufficient to develop a cleft.

We also ran simulations by warping an E10.5 control embryo either along the ‘Neo/Wt’ or ‘Neo/Null’ side of the E11.5 PC1 to visualize how changes in shape over this period affect development (supplementary material Movies 1, 2). These simulations reinforce the idea that the overall growth trajectories of the facial prominences in the two types of embryos were very different. In the embryo developing along the Neo/Wt phenotype, growth and morphogenesis resulted in the whole face moving inward toward the midline. In the Neo/Null example, the maxilla seemed to grow away from the midface, and the tissues that were marked by the landmarks along the base of the nasal pit, which come together in the control, maintained their distance. The abnormal growth of the maxilla in the Neo/Null embryos is also apparent in the raw scans (supplementary material Fig. S2B,C). Specifically, at E11.5, the Neo/Null maxilla was located in a more rostrolateral position, such that, in lateral view, it covered part of the developing eye. Taken together, these data support the idea that the failure of the prominences to fuse and form a normal primary palate is a direct effect of the change in shape.

### The Neo/Null model exhibits subtle changes in growth and Fgf pathway gene expression

**Fig. 3.**
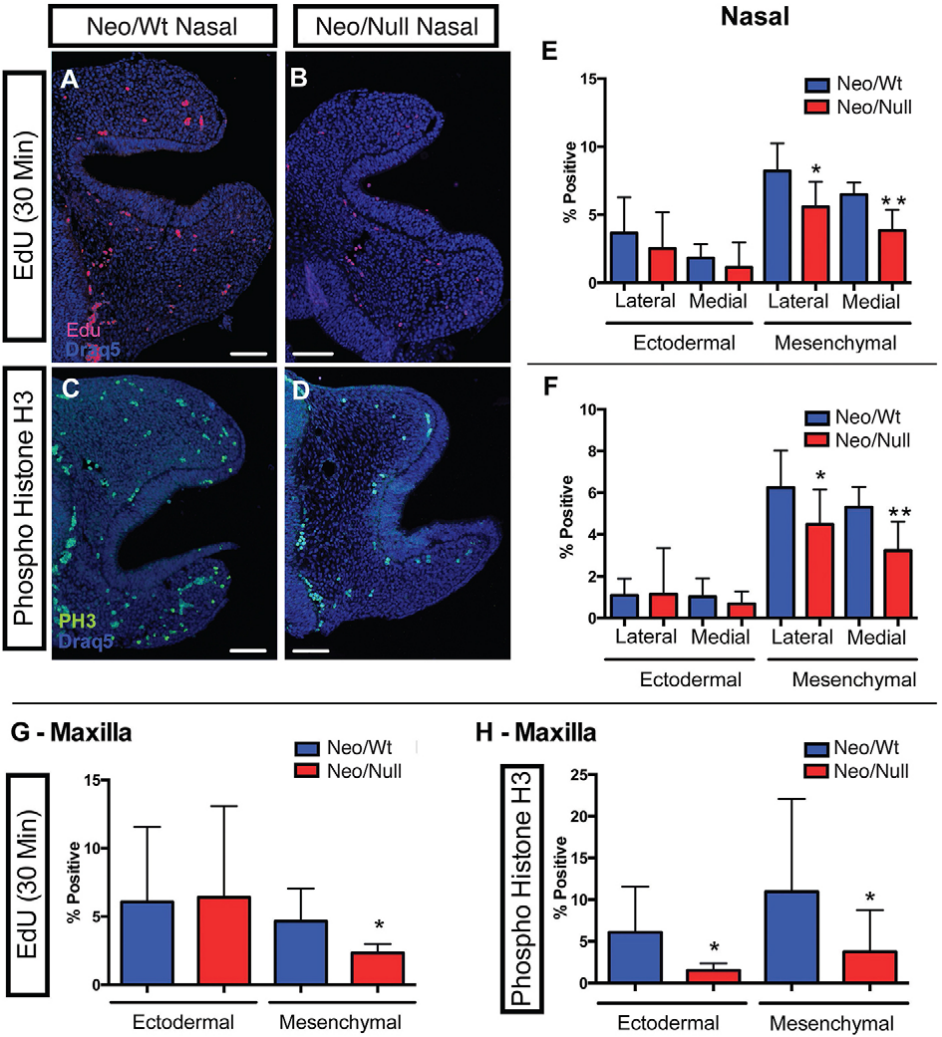
**Reduced cell proliferation in the facial prominences of Neo/Null mice.** Proliferation assessed in sections from the nasal pit of Neo/Wt (A,C) and Neo/Null (B,D) E10.5 embryos using EdU (A,B) or anti-phospho histone H3 (PH3) (C,D) detection. Draq5 was used to show nuclei; scale bars: 75 μm. Distal edge of the nasal pit is to the right. Quantitation of EdU (E,G)- or phospho-histone H3 (F,H)-labeled cells expressed as a percentage of total cells for the nasal (E,F) and maxillary (G,H) prominences. *P<0.05; **P<0.01. Note that any differences in cell density apparent in C and D result from slightly different planes of section rather than inherent differences between the Neo/Wt and Neo/Null models.

It is notable that small differences in local growth rates can produce significant effects on shape (Boehm et al., 2010). Therefore, one explanation for the difference in facial shape and growth trajectory would be a subtle difference in cell proliferation levels between the mutant and the control embryos. To examine proliferation, we utilized EdU, a thymidine analog, to label newly synthesized DNA, as well as an antibody against phospho-histone H3 to identify proliferating cells. Labeled cells were then examined in either the ectoderm or mesenchyme of the prominences. In the mesenchyme, both methods identified consistent decreases in proliferation in the Neo/Null samples for the medial and lateral nasal prominences as well as the maxilla, compared with Neo/Wt controls (Fig. 3A–H). In contrast, proliferation in the ectoderm was not significantly different between Neo/Wt and Neo/Null mutants, with the exception of phospho-histone H3 staining in the maxilla (compare Fig. 3F,H). TUNEL staining did not reveal any significant differences in apoptosis between mutant and control animals, with the exception of the nasal pit ectoderm. In wild-type samples, apoptotic cells were detected where the medial and lateral edges of the nasal pit ectoderm were beginning to fuse. This region of staining, presumably due to apoptosis of the epithelial seam at the fusion point, was not observed in Neo/Null sections, consistent with the failure of fusion between these nasal prominences (supplementary material Fig. S4A,B).

**Fig. 4.**
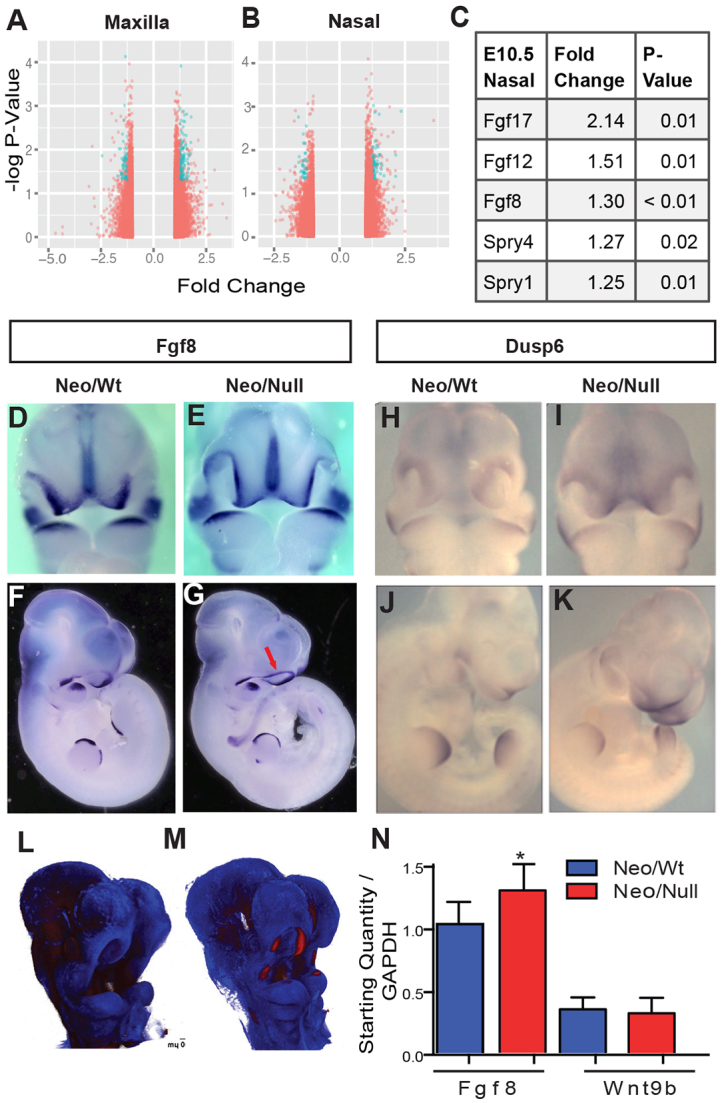
**Alterations in gene expression in Neo/Null mice.** (A,B) Volcano plots from the (A) maxillary and (B) nasal microarrays. Orange shows all values called ‘above absent’, blue all values called ‘present’, with a *P*-value of 0.05 (adjusted) and a fold change >1.25. Positive fold change is increased expression in Neo/Null compared with Neo/Wt. (C) Fold change of Fgf pathway members in Neo/Null E10.5 nasal prominences compared with control embryos. (D–K) *In situ* hybridization for *Fgf8* (D–G) and *Dusp6* (H–K) expression in Neo/Wt (D,F,H,J) and Neo/Null (E,G,I,K) E10.5 embryos showing frontal (D,E,H,I) and lateral (F,G,J,K) views. Arrow in G shows increased *Fgf8* expression at the lateral edge of nasal pit. (L,M) *Fgf8* staining visualized by OPT in E10.5 Neo/Wt (L) and Neo/Null (M) samples. (N) qRT-PCR examination of *Fgf8* and *Wnt9b* transcript levels from E10.5 facial prominences. *P<0.05.

We next compared gene expression profiles in Neo/Null and Neo/Wt nasal and maxillary prominences at E10.5 using microarray analysis. Although differences in shape and proliferation were already apparent at this stage, gene expression changes were moderate (Fig. 4A,B), with less than 300 genes detected as altered by >1.25-fold and few of these altered by >twofold (supplementary material Tables S3, S4). However, analysis of these gene lists using gene set enrichment analysis (GSEA) highlighted enrichment of Fgf pathway members specifically in the nasal prominence (Fig. 4C). Because Fgf signaling has major roles in facial development, this association was further investigated using qRT-PCR and *in situ* hybridization. First, RT-PCR was used on Neo/Wt controls and Neo/Null mutants (Fig. 4N) to examine expression of *Fgf8* as well as *Wnt9b*, an additional gene associated with bilateral CL/P (Jin et al., 2012). In agreement with the microarray data, a consistent ~1.3-fold increase was detected in *Fgf8* expression in the Neo/Null mutants, whereas *Wnt9b* levels were relatively unchanged from controls (Fig. 4N). A slight increase in *Fgf8* expression in the ectoderm of the facial prominences was also revealed by *in situ* analysis coupled with optical projection tomography (OPT) imaging (Fig. 4D–G,L–M). This increase was most notable in the margin of the lateral nasal prominence bordering the nasal pit. The potential upregulation of Fgf signaling was further investigated by examining the expression of pathway targets *Dusp6* and *Mapk1*. *Dusp6*, a negative feedback regulator of the pathway, showed increased expression in the developing facial prominences of Neo/Null mice compared with controls, in contrast to the more comparable expression in the limbs (Fig. 4H–K). However, we did not detect significant changes in the levels of *Mapk1*-encoded total Erk or phospho-Erk by western blot analysis of whole E10.5 facial tissue (supplementary material Fig. S5).

### Reduction of *Fgf8* gene dosage leads to a partial rescue of Neo/Null bilateral clefting

**Fig. 5.**
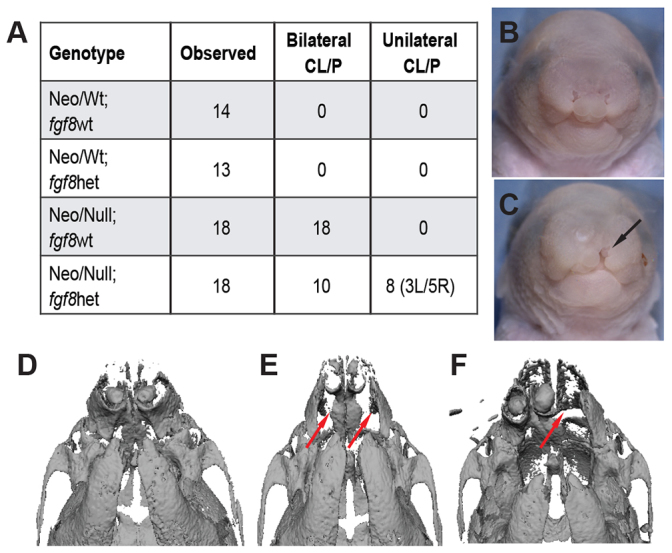
**Reduced *Fgf8* gene dosage leads to a rescue of bilateral cleft lip.** (A) Numbers of various genotypes and phenotypes observed from the *Tfap2a^(neo/neo)^* × *Tfap2a^(+/−)^*;*Fgf8^(+/−)^* crosses (L and R are left- and right-sided cleft, respectively). (B,C) Frontal images of the heads of Neo/Null;*Fgf8*wt (B) and Neo/Null;*Fgf8*het (C) P0 pups. Black arrow shows the unilateral cleft line. (D–F) Ventral view of skulls using μCT for Neo/Wt;*Fgf8*wt (D), Neo/Null;*Fgf8*wt (E) and Neo/Null;*Fgf8*het (F) P0 pups. Red arrows show clefting at points where the premaxilla and the palatal process of the premaxilla should be fused.

**Fig. 6.**
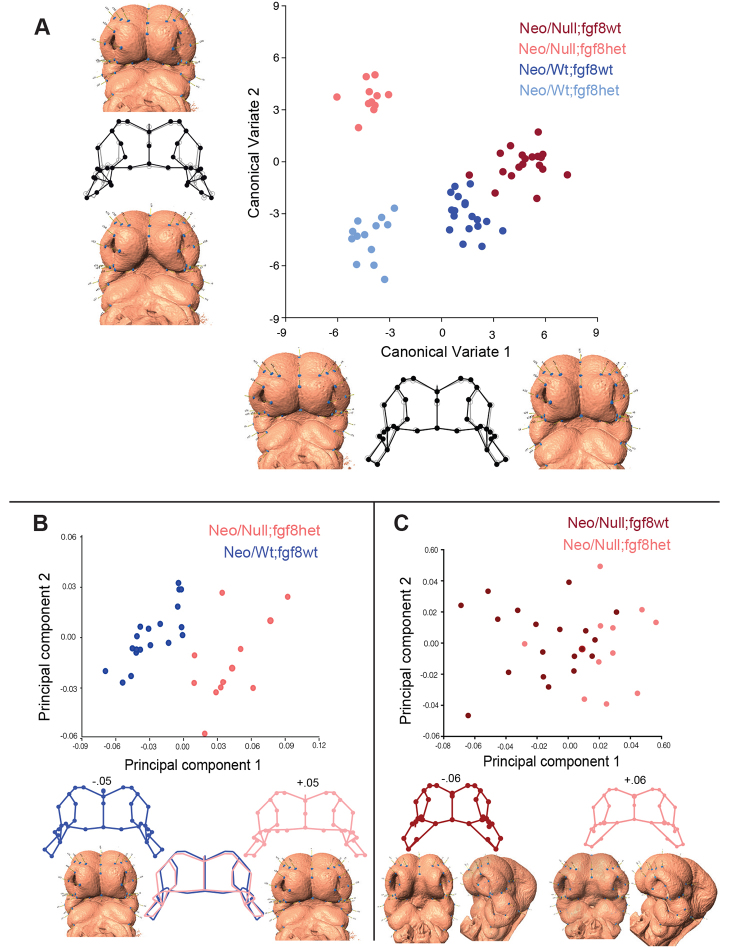
**Morphometric analysis of the genetic interactions between *Tfap2a* and *Fgf8* in facial development.** (A) Morphometric analysis of E10.5 embryos from *Tfap2a^(neo/neo)^* × *Tfap2a^(+/−)^*;*Fgf8^(+/−)^* crosses. CVA showing between-group differences. The wireframes visualize +10 of the axis in black and 0 in gray and the warp images show −6 and +6, respectively, along the axes. (B) PCA of E10.5 Neo/Null;*Fgf8*het compared with Neo/Wt;*Fgf8*wt. Wireframes and warp embryo images show −0.05 and +0.05 along PC1. (C) PCA of E10.5 Neo/Null;*Fgf8*het compared with Neo/Null;*Fgf8*wt. Wireframes and warp embryo images show −0.06 and +0.06 along PC1.

To determine whether changes in Fgf signaling could impact the Neo/Null phenotype, the Neo/Null allelic combination was crossed onto an *Fgf8* heterozygous background (Meyers et al., 1998). This generated four genotypes with various combinations of the *Tfap2a* and *Fgf8* alleles (Fig. 5A). Interestingly, examination of the Neo/Null neonates heterozygous for the *Fgf8* null allele indicated that 8/18 presented with a unilateral cleft primary palate as opposed to the fully penetrant bilateral cleft characteristic of Neo/Null animals with wild-type *Fgf8* gene dosage (Fig. 5A–F). Skeletal organization of ‘rescued’ Neo/Null;Fgf8het mice was assessed by μCT and confirmed a unilateral premaxillary fusion defect (Fig. 5F, red arrow). Note that there was no laterality preference for the ‘rescue’ in Neo/Null;Fgf8het mice, with remaining clefts being detected on either the left or right side of the face. The altered susceptibility to the bilateral facial clefting caused by the reduction of *Fgf8* gene dosage in Neo/Null mice prompted a morphometric analysis of E10.5 embryos to identify early associated shape changes. CVA demonstrated significant between-group differences, with CV1 reflecting differences caused by the *Fgf8* mutation and CV2 reflecting the differences caused by the *Tfap2a* genotype (Fig. 6A). Increases along CV1 represent greater width of the frontal nasal prominence and a more external orientation of the nasal pits. Increases along CV2 increase the width of the nasal pit and change the shape of the maxilla. Intriguingly, the combination of Neo/Null and *Fgf8* null alleles that partially rescued the orofacial clefting did not return the phenotype nearer to the control shape, but resulted in a distinct grouping (Fig. 6A). PCA was next utilized to examine these differences in greater detail (Fig. 6B,C) and indicated that, when compared with the Neo/Wt;Fgf8wt control group, separation along PC1 indicates that the Neo/Null;Fgf8het has a slight widening of the midface but more obviously the nasal pits are somewhat larger and are angled slightly downwards toward the maxilla (Fig. 6B, lower images). Therefore, the frequent rescue of the bilateral clefting in the Neo/Null background caused by reduction of *Fgf8* gene dosage does not occur by simply rectifying the changes observed in the Neo/Null embryos at a similar stage (PC4; Fig. 2B,D and supplementary material Fig. S3). Instead, the reduction of *Fgf8* causes an additional set of changes in the shape of the midface and nasal pits. Furthermore, when these Neo/Null;Fgf8het compound mutants are compared with the Neo/Null;Fgf8wt, we noted a subtle increase in the width of the midface, a lateral pivot of the nasal pit toward the maxilla, and an increase in both the size and width of the nasal pit (Fig. 6C, lower images). These changes would push the nasal pit more into alignment with the maxilla in the Neo/Null;Fgf8het embryos.

**Fig. 7.**
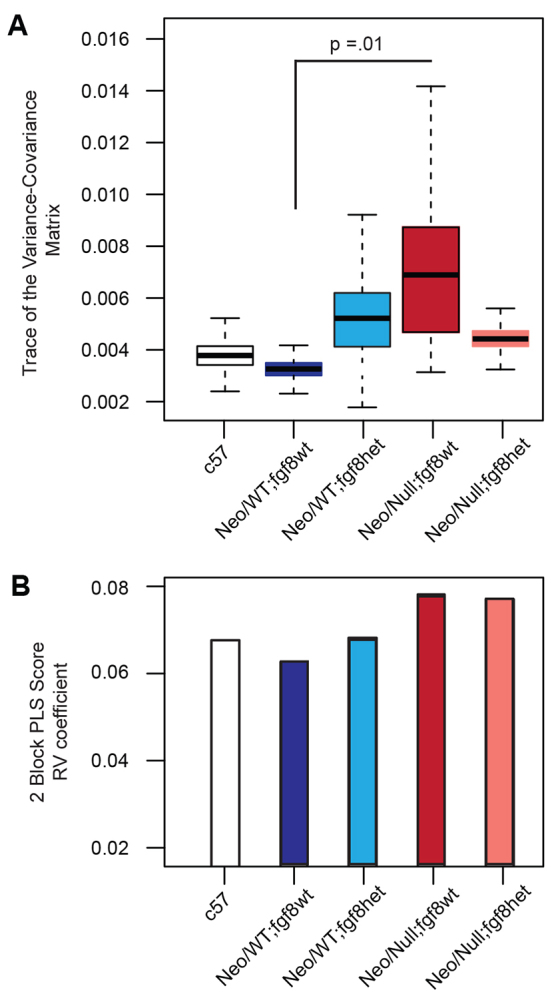
**Analysis of variance and integration of facial morphology associated with *Tfap2a* and *Fgf8* mutations.** (A) Trace of the variance-covariance matrix to measure variation in shape at E10.5. *P*-value was determined based on overlap between the curves based on 1000 repetitions of the analysis. (B) Two-block PLS analysis to determine integration at E10.5 between the maxilla and the nasal prominences, the two regions with the largest differences in shape. Statistics can be found in supplementary material Table S5. The data concerning E10.5 C57Bl/6J is presented as an additional control for background or strain effects and was obtained by re-landmarking scans generated previously (Schmidt et al., 2010).

Previous studies have indicated that a reduction in *Fgf8* dosage alters the mean facial shape relative to wild-type populations and might also lead to an increase in phenotypic variation (Griffin et al., 2013). Such phenotypic variation might influence how an individual mutation affects a population or leads to partial penetrance (Hallgrímsson et al., 2002; Jamniczky and Hallgrímsson, 2009). We therefore investigated whether alterations in *Tfap2a* could also influence phenotypic variance as a potential mechanism for interactions with *Fgf8* gene dosage in modifying orofacial clefting. Specifically, we assessed the variance within the E10.5 dataset, using the trace of the variance covariance matrix, a multivariate measure of variance (Schaefer and Lauder, 1996). In both the Neo/Null;Fgf8wt and Neo/Wt;Fgf8het embryos, the phenotypic variance for shape was significantly increased (Fig. 7A). Surprisingly, however, the combination of these mutant alleles in Neo/Null;Fgf8het embryos did not produce a further increase. In fact, the shape variance in this latter allelic combination was not significantly increased over the controls. Thus, the increase in variance seen with Neo/Null;Fgf8wt and Neo/Wt;Fgf8het seems to be moderated in Neo/Null;Fgf8het. These findings argue against the Neo/Null and *Fgf8* heterozygous changes acting in an additive or synergistic manner on the variance, and instead indicate that the combination of these mutations unexpectedly dampens the amount of variance.

Another method for observing the interaction between shape changes is to examine how different parts of the face are integrated with one another, or simply to identify how much changes in one region correlate with changes in another. Observations of integration can also determine how underlying gene expression changes can affect shape. Therefore, a two-block partial least squares regression (PLS) analysis was used to examine correlation of shape changes between the nasal processes and the maxilla between the various genotypes (Fig. 7B and supplementary material Table S5). This analysis indicated a very high degree of integration of shape differences between the prominences in the various models. The vectors describing the directions of landmark displacements that correspond to the covariances between the nasal and maxillary processes were also significantly correlated across the samples (*P*-values in supplementary material Table S5). This indicates that both the pattern and magnitude of integration among the processes are similar across genotypes and further suggests that the same processes that increase variance are also linked to overall structural changes within the face.

## DISCUSSION

In this study, we describe the derivation of a new mouse model displaying fully penetrant bilateral CL/P that is caused by an insertional mutation in an intron of *Tfap2a*. These studies further strengthen the importance of the AP-2 gene family, particularly *Tfap2a*, in facial development. Genetic manipulation of mouse *Tfap2a* results in multiple defects in development and function of the skull and face (Brewer and Williams, 2004; Nelson and Williams, 2004; Nottoli et al., 1998; Pontoriero et al., 2008; Schorle et al., 1996; Zhang et al., 1996). Moreover, recent analyses have identified mutations in *TFAP2A* as the cause of human BOFS, which is characterized in part by orofacial clefts of varying severity (Milunsky et al., 2008; Milunsky et al., 2011). AP-2α has also been linked to human orofacial clefting defects via its regulation of target genes, including *p63* and *IRF6* (Ferretti et al., 2011; Rahimov et al., 2008; Wang et al., 2013). Here, we have shown that, as the level of functional *Tfap2a* transcripts are decreased, presumably below a certain threshold, there are changes in Fgf signaling, prominence growth and facial shape that are associated with orofacial clefting.

The primary conclusion from our study is that the failure of facial fusion in the Neo/Null model is due to changes in the growth and morphogenesis of the facial prominences. Using μCT and geometric morphometrics, differences in facial shape between Neo/Null and control embryos were apparent by E10.25, and the morphologies of the facial prominences became highly divergent by E11.5. Analysis of the relative movement of the components of the face over time in control embryos indicates that all the facial prominences normally wrap around the mediolateral axis and converge towards the midline. However, in Neo/Null embryos, the lateral edge of the nasal pit flares outward instead of growing in toward the center and the maxillary prominence is also projected laterally. These alterations from the normal morphogenetic pathway prevent apposition and fusion of these facial prominences. Therefore, in common with some forms of CP, CL/P develops in this model because the prominences are not aligned properly for fusion. Previous studies in chick have indicated that directional differences in cell proliferation can affect shape and overall growth trajectories of the facial prominences (Li et al., 2013b). Similarly, in the Neo/Null mouse model, we show that one consequence of decreased *Tfap2a* levels is a reduced rate of proliferation in the mesenchyme of the nasal and maxillary prominences, which would presumably lead to a change in the overall growth and, potentially, the shape of the nasal pit and midface.

A secondary conclusion from these analyses is that neural tube closure defects correlate with differences in the shape of the face from very early time points. A subset of the Neo/Null mice developed exencephaly and, by E9.5, this group could be clearly distinguished on the basis of facial shape from other embryos with closed or delayed neural tube closure. There are several possible explanations for the effect of aberrant neural tube closure on face development. One hypothesis suggested by the μCT imagery is that facial defects are linked with defective morphogenesis of the telencephalon. In particular, failure to close the neural tube leads to a much narrower telencephalon and, based on data indicating that the forebrain acts as a major signaling center for midfacial development (Gongal et al., 2011; Hu and Marcucio, 2009), this would alter regulatory crosstalk between these two regions. Alternatively, the neural tube defect might lead to abnormal formation or migration of the neural crest that could limit the population of mesenchymal cells available for face formation. Whatever mechanism is responsible for the distinct facial morphology of the exencephalic Neo/Null mice, the overall result suggests that understanding the etiology of CL/P in humans will be complicated if this pathology is also accompanied by a cranial neural tube closure defect. Ultimately, detailed morphometric analysis of mice with an isolated cranial neural tube closure defect will be necessary to determine whether the morphological changes caused by neural tube closure and facial clefting are synergistic or independent events.

Previous morphometric studies of orofacial clefting in mouse have focused on partially penetrant clefting models, particularly the A/WySn and Cl/Fr strains. The A/WySn mutation has been linked to mutation of *Wnt9b*, and a total loss of *Wnt9b* generates a clefting frequency of 50–100% depending on background (Jin et al., 2012; Juriloff and Harris, 2008; Juriloff et al., 2006). Cl/Fr and A/WySn strains have considerable within-group variation, which might correlate with the partially penetrant nature of their mutant facial phenotypes, and it has not been possible to identify definitive early changes that will result in CL/P. Nevertheless, these studies are pertinent to the shape changes observed in Neo/Null mice. In the A/WySn model, the facial prominences are less well developed overall compared with controls at a similar embryonic stage, and the shape difference seems to be concentrated in the maxilla. In common with the Neo/Null model, the hypoplastic development might be due to decreased cell proliferation in the mesenchyme, because this also occurs in the *Wnt9b*-null mouse (Jin et al., 2012). The Cl/Fr strain, which has been studied using 2D morphometric methods, shows shape differences primarily in the nasal pit (Parsons et al., 2008; Young et al., 2007).

Neo/Null is the first fully penetrant bilateral clefting model examined using morphometric methods and combines the separate aspects of these other two models, with simultaneous morphological differences in both the maxilla and nasal pit. We propose that the combination of these two potentially independent shape changes distinguishes the fully penetrant CL/P Neo/Null phenotype from the two previously studied partially penetrant models. Taken together, though, the morphometric data obtained from these three models of CL/P indicate that they each exhibit modest changes in the shape and growth direction of the facial prominences. Such alterations would prevent these structures from being in the correct positions at the correct times, thus precluding normal fusion.

Despite the similarities with the A/WySn model noted above, we did not detect differences in the expression of *Wnt9b* in the Neo/Null mice. Indeed, overall gene expression changes between Neo/Null and controls were limited in both number and fold change despite the resulting severe facial pathology. These findings indicate that reduced *Tfap2a* levels do not cause drastic changes in the expression of a specific set of gene targets, but instead might cause more subtle differences in the overall gene regulatory network controlling facial development. We note that similar conclusions were obtained from a study regarding the influence of the *Myc* transcription factor on mouse facial development (Uslu et al., 2014). However, we did find evidence of alterations in Fgf signaling in the Neo/Null model, including a modest upregulation of *Fgf8*, *Fgf12* and *Fgf17* expression as well as of the Fgf target *Dusp6*. *Fgf8* has proven roles in the regulation of facial shape, facial development and midfacial integration in multiple species (reviewed in Dorey and Amaya, 2010; Griffin et al., 2013; Inman et al., 2013). Previous studies have also shown that modulation of Fgf signaling through altering *Fgf8* gene dosage can either exacerbate or ameliorate mouse craniofacial defects (Inman et al., 2013; Tabler et al., 2013). Crucially, we demonstrate that modifying *Fgf8* gene dosage can also rescue clefts of the primary palate, in this instance revealing a genetic interaction between *Fgf8* and *Tfap2a*. This finding expands the repertoire of genetic interactions involving *Fgf8* that alter facial development to include *Foxc1*, *Fuz* and *Tfap2a*, suggesting that *Fgf8* might act in concert with multiple developmental regulatory programs to modify face formation (Inman et al., 2013; Tabler et al., 2013).

As noted above, in the Neo/Null model, we observe changes in the shape of both the nasal and maxillary prominences that places them in more lateral positions. The shape change in the *Fgf8* heterozygous mice instead affects only the nasal pit, which is however also tilted outwards. It seems, therefore, that the Neo/Null clefting defect could be rescued when combined with an *Fgf8* mutation because the nasal pit is positioned more laterally and proximally, allowing an increased area for interaction, and therefore fusion, with the maxilla. Thus, the alteration in shape when *Fgf8* gene dosage is reduced does not seem to directly ‘reverse’ or ‘undo’ the shape change in the Neo/Null mutation; rather, it provides a compensatory change that facilitates fusion. The observation that manipulation of Fgf signaling can rescue aspects of bilateral CL/P in the Neo/Null model might also have wider significance for understanding and treating orofacial clefting in both mouse and human. In future, it would valuable to determine if genetic or pharmaceutical alteration of Fgf signaling could alter the severity and frequency of clefting in other mouse models that exhibit this pathology.

In addition to changes in mean shape, we also observed changes in shape variance in our model, which could be pertinent to the evolutionary constraints that act upon facial morphology. Recent studies have indicated that only a limited number of facial shapes are developmentally tolerated across a wide array of species (Young et al., 2014). In support of this theory, human studies demonstrate differences in the facial shape of control groups compared with those of unaffected relatives of individuals with CL/P, suggesting that morphology might predispose individuals to clefting (Weinberg et al., 2009; Weinberg et al., 2008). Phenotypic heterogeneity is a common feature of human birth defects, including CL/P (Milunsky et al., 2011). The extent to which increased shape variance, caused by underlying mutations, contributes to this heterogeneity remains virtually unknown. In mouse, CL/P has been hypothesized to occur at extremes of normal variation of facial shape (Juriloff and Trasler, 1976; Trasler, 1968). Therefore, increased variance might also contribute to both penetrance and expressivity of human CL/P, because this might push some individuals, or sides within individuals, over a theoretical threshold beyond which normal lip fusion fails (Parsons et al., 2008). It is therefore intriguing that alteration of *Tfap2a* and *Fgf8* can influence variance (this study) (Griffin et al., 2013). Moreover, the observation that the combination of mutations in these two genes dampens the variance caused by mutations affecting only a single gene is intriguing and suggests interacting pathways. Further studies are warranted to determine the mechanistic links between *Tfap2a* and Fgf signaling as well as a possible wider role for variance in facial shape and orofacial clefting.

In summary, analysis of the robust Neo/Null orofacial clefting model provides the first direct evidence in mouse demonstrating that facial shape changes directly interfere with prominence fusion. We also note that the overall shape and gene expression changes in the Neo/Null model are relatively modest and yet lead to drastic morphological consequences. Based on these observations, we speculate that the process of facial fusion is exquisitely sensitive to subtle changes in the shape of the facial prominences, which would render this developmental process prone to genetic and environmental perturbation consistent with the frequent occurrence of human CL/P birth defects. Our data also suggest that combinations of effects across multiple pathways can influence facial shape and the generation of dysmorphology in ways that are complex and not easily predicted. Recently, additional mouse models with fully penetrant CL/P have been identified, including mutations involving *Lrp6*, *Pbx* and *Bmpr1a* (Das and Crump, 2012; Ferretti et al., 2011; Liu et al., 2005; Song et al., 2009; Wang et al., 2013). Morphometric analysis of these additional models would determine whether they also involve similar subtle changes in growth trajectories of the prominences, or whether further mechanisms, such as a failure of fusion following normal apposition of the prominences, can also account for this widespread human pathology.

## MATERIALS AND METHODS

### Breeding and genotyping

Mice were maintained on an outbred Black Swiss background (Charles River, Wilmington, MA). To obtain Neo/Null embryos, female Neo homozygote animals [*Neoflox* as described previously (Brewer et al., 2004)] were crossed with *Tfap2a*^+/−^ male mice (Zhang et al., 1996) to yield 50% Neo/Null and 50% Neo/Wt. To obtain Neo/Null embryos that were also heterozygous for *Fgf8*, female Neo homozygote animals were crossed with *Tfap2a^+/−^*; *Fgf8^+/−^* male mice. The *Fgf8* null allele was derived by crossing the *Fgf8flox* line, *Fgf8^tm1.3Mrt^*, with β-actin Cre transgenic mice (Meyers et al., 1998), and then crossing back to Black Swiss mice to remove the β-actin Cre transgene. Females were examined in the morning for presence of a vaginal plug, and the presence of a plug was designated E0.5. Genomic DNA for genotyping was derived from tail biopsies or from the embryonic yolk sac and genotyping was performed as previously reported (Brewer et al., 2004; Meyers et al., 1998; Zhang et al., 1996). All animal experiments were performed in accordance with protocols approved by the University of Colorado Denver (UCD) Animal Care and Usage Committee.

### Microarray

E10.5 facial tissue was microdissected into separate nasal and maxillary prominence fractions. Samples were pooled to generate sufficient tissue for three independent biological replicates. Microarray analyses were then performed in triplicate using these pooled samples as described (Feng et al., 2009). Briefly, total RNA was extracted using Trizol (Life Technologies, Carlsbad, CA), then purified using the RNAeasy MiniKit (Qiagen, Venlo, The Netherlands). Microarray analyses were carried out by the UCD Gene Expression Core Facility. *In vitro* transcription (IVT) was performed to generate biotin-labeled cRNA using an RNA Transcript Labeling Kit (Enzo Inc., Farmingdale, NY). Biotin-labeled cRNA was purified using an RNeasy affinity column (Qiagen) and the cRNA was fragmented and analyzed on the Agilent 2100 Bioanalyzer (Agilent Technologies, Palo Alto, CA). cRNA was hybridized to Affymetrix GeneChipH Mouse430 2.0 microarrays. Processing was performed in the GeneChipH Operating Software (Affymetrix) as discussed previously (Feng et al., 2009) using a scaling factor to bring the average intensity for all probes on the array to the same target intensity value, allowing samples to be compared across arrays. All data from our analysis are available via GEO (GSE60058; http://www.ncbi.nlm.nih.gov/geo/query/acc.cgi?acc=GSE60058). Analysis of gene representations was performed using GSEA (Subramanian et al., 2005).

### Reverse-transcription PCR and real-time PCR

RNA was isolated from microdissected E10.5 faces through a standard Trizol protocol (Life Technologies). cDNA was made using the SuperScript III First-Strand Synthesis Kit for RT-PCR (Life Technologies) using 1 μg RNA/10 μl reaction. Three biological replicates were used for all analyses. For RT-PCR, the following primers were used: 5F: 5′-GAGACGTAAAGCTGCCAACGTTACCCTCCTC-3′; 7R: 5′-CATGGGAGATGAGGTTGAAGTGGGTCAAGC-3′; PGKup: 5′-GCGCCTCCCCTACCCGGTAGAATTGGCCGCG-3′. For real-time PCR, pre-validated probes and primers were used to examine *Tfap2a* levels targeting exons 2–3 and 6–7 (IDT PrimeTime assay Mm.PT.56a.1580695 and 12995135, Integrated DNA Technologies, Coralville, IA) as well as *Fgf8* (TaqMan gene expression assay Mm00438922_m1, Applied Biosystems, San Francisco, CA) and *Wnt9b* (TaqMan gene expression assay Mm00457102_m1). *Gapdh* was used as a housekeeping control for all assays (ABI, TaqMan gene expression assay Mm99999915_g1). The TaqMan Gene Expression Master Mix was used and samples were run on a BioRad MyCycler. Each biological replicate was run in duplicate. Quantification was performed by comparison to a standard curve.

### *In situ* hybridization and optical projection tomography (OPT)

*In situ* hybridization was performed as previously described (Feng et al., 2009). Briefly, dissected embryos were fixed overnight in 4% paraformaldehyde in phosphate buffered saline (PBS), dehydrated through a PBS/methanol gradient series (75% PBS/25% methanol, 50/50, 25/75) and then stored in 100% methanol at −20°C. At the start of the protocol embryos were taken back through the series into PBST (0.01% Tween 20). Embryos were incubated in 10 μg/ml proteinase K for 20 minutes, re-fixed in 4% paraformaldehyde + 0.1% glutaraldehyde for 20 minutes, then pre-hybridized in hybridization buffer (50% Formamide, 1.3×SSC, 50 μg/ml Yeast tRNA, 100 μg/ml Heparin, 0.2% Tween 20, 0.5% CHAPS, 5 mM EDTA) for 3 hours at 70°C. Embryos were then incubated in fresh hybridization buffer containing dig-UTP-labeled probe overnight at 70°C. Next, embryos were washed in hybridization buffer then in Maleic acid buffered saline with 0.05% Tween 20 (MABT). Embryos were then blocked in 20% sheep serum with 2% blocking reagent (Roche, Penzberg, Germany). Probe was detected using an anti-digoxigenin antibody (Roche) diluted 1:2000 in the blocking reagent and incubated overnight at 4°C. Following 2 days of rinsing in MABT and alkaline phosphatase buffer, signal was developed in BM Purple (Roche).

For OPT, after completion of the whole-mount *in situ* hybridization procedure, embryos were embedded in 1% low-melt agarose (Life Technologies) then cleared in methanol for 24 hours followed by overnight in a mixture of 1 benzyl alcohol:2 benzyl benzoate. Cleared embryos were imaged on the Bioptonics 3000 scanner and images reconstructed using the SkyScan software.

### Bone and cartilage staining

E18.5 fetuses were sacrificed and stained as previously described (Brewer and Williams, 2004). Briefly, fetuses were incubated 2 days in 100% ethanol, 2 days in acetone, 1 week in dye solution (0.1% Alizarin red and 0.3% Alcian blue in 70% ethanol) then cleared through a series of potassium hydroxide (2%) and glycerol.

### Western blot

Phosphatase inhibitor (Pierce cat # 8868, Rockford, IL)-treated lysates from E10.5 dissected faces were separated by 10% denaturing PAGE then transferred to a nitrocellulose membrane, blocked 1 hour in 5% BSA, then probed for phospho-Erk (Cell Signaling Technology #4370, Danvers, MA), total Erk1 (Santa Cruz Biotech SC-94, Santa Cruz, CA) and Erk2 (Santa Cruz SC-153).

### Cell proliferation and apoptosis

Pregnant females were injected with 200 μg of Click-it EdU (BrdU analog, Life Technologies) 30 minutes prior to sacrifice. Embryos were dissected at E10.5, immediately fixed in 4% paraformaldehyde in PBS overnight, dehydrated through an ethanol series (25%, 50%, 70% overnight, 100% 1 hour), then processed through a xylene and paraffin series (100% xylene: 50% xylene + 50% paraffin; 100% paraffin), embedded in paraffin and cut as 8-μm sections. Only embryos with closed neural tubes were selected for analysis. Sections were dewaxed and rehydrated and then imaged following the manufacturer’s instructions omitting the Hoechst blue. Following the Click-it chemistry using the 594 fluorophore, slides were blocked for 1 hour in Cell Signaling block buffer, and incubated overnight with a rabbit anti-phospho histone H3 antibody (Cell Signaling Technology #9701). Cells were imaged following detection using goat anti-rabbit Alexa Fluor 488 secondary antibody (Cell Signaling Technology #4412). For TUNEL, alternate sections were selected and stained using the ApopTag fluorescein kit following the manufacturer’s instructions, except that sections were permeabilized according to the Click-it kit (0.1% Triton-X for 10 minutes) (Millipore #7160, Millipore, Billerica, MA). All sections were counterstained with Hoechst blue and Draq5 (Cell Signaling Technology #4084). Images were generated on a Leica SP5 confocal microscope and nuclei were hand counted on ImageJ. Three embryos per condition were counted and three sections were analyzed per animal.

### Morphometrics

Embryos were dissected between E9.5 and E11.5, and immediately fixed in 4% paraformaldehyde and 5% glutaraldehyde as described previously (Schmidt et al., 2010). These time points were chosen because they cover the majority of craniofacial development prior to fusion of the primary palate. Fixed embryos were immersed in Cysto ConRay II^®^ (iothalamate meglumine) contrast agent for 1 hour then scanned on a μCT35 scanner to a 3.5 or 7.5 μm resolution. For the analysis of skeletons, neonates were fixed in 4% paraformaldehyde and scanned at 7.5 μm resolution. Landmarking was performed in MeshLab (http://meshlab.sourceforge.net). Landmarks represent Bookstein type 1 and 2 landmarks (Bookstein, 1997; Zelditch et al., 2012) and are similar to the lab-established landmarks (Parsons et al., 2011) with additional landmarks around the maxillary prominence. Additionally, about 20 previously generated E10.5 C57Bl/6J scans from Schmidt et al. (Schmidt et al., 2010) were re-landmarked and used as an additional control for background or strain effects. All analyses, except scaled variance of the eigenvectors and trace of the eigenvectors, were performed in MorphoJ 1.05b (Klingenberg, 2011). Procrustes superimposition was used to remove the effects of alignment, rotation or scale. All embryos were divided into groups based on tail somite numbers. Tail somites (Som) were counted from the tail tip to the base of the forelimb. 12–21 Som were classified as E9.25–9.75, 23–35 as E10.25–10.75, and 40–50 as E11.25–11.75. Data were regressed against tail somites using group centered regressions and only within each age-based group, because it is easier to regress out age if the age-related changes are smaller. All further analysis was performed on the residuals from the regression. PCA was used to view spread of data and identify major axes of variance among individuals. CVA was used to determine major axes describing among-group variation. Procrustes distance and Procrustes permutation tests were used to determine *P*-values for between-group differences. The two-block PLS was run using block 1 as the nasal prominence landmarks and block 2 as the maxillary prominence landmarks, and the compare vector test was used to examine differences between groups. Movies and mesh warp images were created using landmark IDAV (Wiley et al., 2005), which uses 3D splines to warp one 3D image to a new set of 3D coordinates. Screen shots were then taken of the new 3D surface at various angles. For movies, a screen shot was taken for every 2% difference and then images were loaded into iMovie (Apple) to create the animations. Scaled variance of the eigenvalues and trace of the eigenvectors were performed as reported previously using the statistical software R (http://www.r-project.org) (Hallgrímsson et al., 2009).

## Supplementary Material

Supplementary Material

## References

[b1-0080031] Boehm B. Westerberg H. Lesnicar-Pucko G. Raja S. Rautschka M. Cotterell J. Swoger J. Sharpe J. (2010). The role of spatially controlled cell proliferation in limb bud morphogenesis. PLoS Biol. 8, e1000420. 20644711 10.1371/journal.pbio.1000420 PMC2903592

[b2-0080031] Bookstein F. L. (1997). Morphometric Tools for Landmark Data. Cambridge: Cambridge University Press.

[b3-0080031] Brewer S. Williams T. (2004). Loss of AP-2alpha impacts multiple aspects of ventral body wall development and closure. Dev. Biol. 267, 399–417. 15013802 10.1016/j.ydbio.2003.11.021

[b4-0080031] Brewer S. Feng W. Huang J. Sullivan S. Williams T. (2004). Wnt1-Cremediated deletion of AP-2alpha causes multiple neural crest-related defects. Dev. Biol. 267, 135–152. 14975722 10.1016/j.ydbio.2003.10.039

[b5-0080031] Brugmann S. A. Tapadia M. D. Helms J. A. (2006). The molecular origins of species-specific facial pattern. Curr. Top. Dev. Biol. 73, 1–42. 16782454 10.1016/S0070-2153(05)73001-5

[b6-0080031] Bush J. O. Jiang R. (2012). Palatogenesis: morphogenetic and molecular mechanisms of secondary palate development. Development 139, 231–243. 22186724 10.1242/dev.067082 PMC3243091

[b7-0080031] Carroll T. J. Park J.-S. Hayashi S. Majumdar A. McMahon A. P. (2005). Wnt9b plays a central role in the regulation of mesenchymal to epithelial transitions underlying organogenesis of the mammalian urogenital system. Dev. Cell 9, 283–292. 16054034 10.1016/j.devcel.2005.05.016

[b8-0080031] Chai Y. Maxson R. E. Jr (2006). Recent advances in craniofacial morphogenesis. Dev. Dyn. 235, 2353–2375. 16680722 10.1002/dvdy.20833

[b9-0080031] Cooper W. J. Albertson R. C. (2008). Quantification and variation in experimental studies of morphogenesis. Dev. Biol. 321, 295–302. 18619435 10.1016/j.ydbio.2008.06.025

[b10-0080031] Das A. Crump J. G. (2012). Bmps and id2a act upstream of Twist1 to restrict ectomesenchyme potential of the cranial neural crest. PLoS Genet. 8, e1002710. 22589745 10.1371/journal.pgen.1002710 PMC3349740

[b11-0080031] Dorey K. Amaya E. (2010). FGF signalling: diverse roles during early vertebrate embryogenesis. Development 137, 3731–3742. 20978071 10.1242/dev.037689 PMC3747497

[b12-0080031] Fan Z. Yamaza T. Lee J. S. Yu J. Wang S. Fan G. Shi S. Wang C.-Y. (2009). BCOR regulates mesenchymal stem cell function by epigenetic mechanisms. Nat. Cell Biol. 11, 1002–1009. 19578371 10.1038/ncb1913 PMC2752141

[b13-0080031] Feng W. Leach S. M. Tipney H. Phang T. Geraci M. Spritz R. A. Hunter L. E. Williams T. (2009). Spatial and temporal analysis of gene expression during growth and fusion of the mouse facial prominences. PLoS ONE 4, e8066. 20016822 10.1371/journal.pone.0008066 PMC2789411

[b14-0080031] Ferretti E. Li B. Zewdu R. Wells V. Hébert J. M. Karner C. Anderson M. J. Williams T. Dixon J. Dixon M. J. (2011). A conserved Pbx-Wnt-p63-Irf6 regulatory module controls face morphogenesis by promoting epithelial apoptosis. Dev. Cell 21, 627–641. 21982646 10.1016/j.devcel.2011.08.005 PMC3199312

[b15-0080031] Gongal P. A. French C. R. Waskiewicz A. J. (2011). Aberrant forebrain signaling during early development underlies the generation of holoprosencephaly and coloboma. Biochim. Biophys. Acta 1812, 390–401. 20850526 10.1016/j.bbadis.2010.09.005

[b16-0080031] Griffin J. N. Compagnucci C. Hu D. Fish J. Klein O. Marcucio R. Depew M. J. (2013). Fgf8 dosage determines midfacial integration and polarity within the nasal and optic capsules. Dev. Biol. 374, 185–197. 23201021 10.1016/j.ydbio.2012.11.014 PMC4086262

[b17-0080031] Gritli-Linde A. (2007). Molecular control of secondary palate development. Dev. Biol. 301, 309–326. 16942766 10.1016/j.ydbio.2006.07.042

[b18-0080031] Gritli-Linde A. (2010). p63 and IRF6: brothers in arms against cleft palate. J. Clin. Invest. 120, 1386–1389. 20424318 10.1172/JCI42821 PMC2860914

[b19-0080031] Hallgrímsson B. Willmore K. Hall B. K. (2002). Canalization, developmental stability, and morphological integration in primate limbs. Am. J. Phys. Anthropol. Suppl. 35, 131–158. 12653311 10.1002/ajpa.10182 PMC5217179

[b20-0080031] Hallgrímsson B. Jamniczky H. Young N. M. Rolian C. Parsons T. E. Boughner J. C. Marcucio R. S. (2009). Deciphering the palimpsest: studying the relationship between morphological integration and phenotypic covariation. Evol. Biol. 36, 355–376. 23293400 10.1007/s11692-009-9076-5 PMC3537827

[b21-0080031] Hester M. Thompson J. C. Mills J. Liu Y. El-Hodiri H. M. Weinstein M. (2005). Smad1 and Smad8 function similarly in mammalian central nervous system development. Mol. Cell. Biol. 25, 4683–4692. 15899870 10.1128/MCB.25.11.4683-4692.2005 PMC1140628

[b22-0080031] Hu D. Marcucio R. S. (2009). A SHH-responsive signaling center in the forebrain regulates craniofacial morphogenesis via the facial ectoderm. Development 136, 107–116. 19036802 10.1242/dev.026583 PMC2685963

[b23-0080031] Inman K. E. Purcell P. Kume T. Trainor P. A. (2013). Interaction between Foxc1 and Fgf8 during mammalian jaw patterning and in the pathogenesis of syngnathia. PLoS Genet. 9, e1003949. 24385915 10.1371/journal.pgen.1003949 PMC3868537

[b24-0080031] Jamniczky H. A. Hallgrímsson B. (2009). A comparison of covariance structure in wild and laboratory muroid crania. Evolution 63, 1540–1556. 19210537 10.1111/j.1558-5646.2009.00651.x

[b25-0080031] Jin Y. R. Han X. H. Taketo M. M. Yoon J. K. (2012). Wnt9b-dependent FGF signaling is crucial for outgrowth of the nasal and maxillary processes during upper jaw and lip development. Development 139, 1821–1830. 22461561 10.1242/dev.075796 PMC3328181

[b26-0080031] Juriloff D. M. Harris M. J. (2008). Mouse genetic models of cleft lip with or without cleft palate. Birth Defects Res. A Clin. Mol. Teratol. 82, 63–77. 18181213 10.1002/bdra.20430

[b27-0080031] Juriloff D. M. Trasler D. G. (1976). Test of the hypothesis that embryonic face shape is a causal factor in genetic predisposition to cleft lip in mice. Teratology 14, 35–41. 960009 10.1002/tera.1420140106

[b28-0080031] Juriloff D. M. Harris M. J. McMahon A. P. Carroll T. J. Lidral A. C. (2006). Wnt9b is the mutated gene involved in multifactorial nonsyndromic cleft lip with or without cleft palate in A/WySn mice, as confirmed by a genetic complementation test. Birth Defects Res. A Clin. Mol. Teratol. 76, 574–579. 16998816 10.1002/bdra.20302

[b29-0080031] Klingenberg C. P. (2010). Evolution and development of shape: integrating quantitative approaches. Nat. Rev. Genet. 11, 623–635. 20697423 10.1038/nrg2829

[b30-0080031] Klingenberg C. P. (2011). MorphoJ: an integrated software package for geometric morphometrics. Mol. Ecol. Resour. 11, 353–357. 21429143 10.1111/j.1755-0998.2010.02924.x

[b31-0080031] Kohlbecker A. Lee A. E. Schorle H. (2002). Exencephaly in a subset of animals heterozygous for AP-2alpha mutation. Teratology 65, 213–218. 11967920 10.1002/tera.10037

[b32-0080031] Li H. Sheridan R. Williams T. (2013a). Analysis of TFAP2A mutations in Branchio-Oculo-Facial Syndrome indicates functional complexity within the AP-2α DNA-binding domain. Hum. Mol. Genet. 22, 3195–3206. 23578821 10.1093/hmg/ddt173 PMC3723307

[b33-0080031] Li X. Young N. M. Tropp S. Hu D. Xu Y. Hallgrímsson B. Marcucio R. S. (2013b). Quantification of shape and cell polarity reveals a novel mechanism underlying malformations resulting from related FGF mutations during facial morphogenesis. Hum. Mol. Genet. 22, 5160–5172. 23906837 10.1093/hmg/ddt369 PMC3842176

[b34-0080031] Liu W. Sun X. Braut A. Mishina Y. Behringer R. R. Mina M. Martin J. F. (2005). Distinct functions for Bmp signaling in lip and palate fusion in mice. Development 132, 1453–1461. 15716346 10.1242/dev.01676

[b35-0080031] Martínez-Abadías N. Mitteroecker P. Parsons T. E. Esparza M. Sjøvold T. Rolian C. Richtsmeier J. T. Hallgrímsson B. (2012). The developmental basis of quantitative craniofacial variation in humans and mice. Evol. Biol. 39, 554–567. 23226904 10.1007/s11692-012-9210-7 PMC3514712

[b36-0080031] Martínez-Abadías N. Holmes G. Pankratz T. Wang Y. Zhou X. Jabs E. W. Richtsmeier J. T. (2013). From shape to cells: mouse models reveal mechanisms altering palate development in Apert syndrome. Dis. Model. Mech. 6, 768–779. 23519026 10.1242/dmm.010397 PMC3634659

[b37-0080031] McIntyre G. T. Mossey P. A. (2002). The craniofacial morphology of the parents of children with orofacial clefting: a systematic review of cephalometric studies. J. Orthod. 29, 23–29. 11907306 10.1093/ortho/29.1.23

[b38-0080031] Meyers E. N. Lewandoski M. Martin G. R. (1998). An Fgf8 mutant allelic series generated by Cre- and Flp-mediated recombination. Nat. Genet. 18, 136–141. 9462741 10.1038/ng0298-136

[b39-0080031] Milunsky J. M. Maher T. A. Zhao G. Roberts A. E. Stalker H. J. Zori R. T. Burch M. N. Clemens M. Mulliken J. B. Smith R. (2008). TFAP2A mutations result in branchio-oculo-facial syndrome. Am. J. Hum. Genet. 82, 1171–1177. 18423521 10.1016/j.ajhg.2008.03.005 PMC2427243

[b40-0080031] Milunsky J. M. Maher T. M. Zhao G. Wang Z. Mulliken J. B. Chitayat D. Clemens M. Stalker H. J. Bauer M. Burch M. (2011). Genotypephenotype analysis of the branchio-oculo-facial syndrome. Am. J. Med. Genet. A 155, 22–32. 21204207 10.1002/ajmg.a.33783

[b41-0080031] Mossey P. A. Modell B. (2012). Epidemiology of oral clefts 2012: an international perspective. Front Oral Biol 16, 1–18. 22759666 10.1159/000337464

[b42-0080031] Nakasima A. Ichinose M. (1983). Characteristics of craniofacial structures of parents of children with cleft lip and/or palate. Am. J. Orthod. 84, 140–146. 6576638 10.1016/0002-9416(83)90178-1

[b43-0080031] Nelson D. K. Williams T. (2004). Frontonasal process-specific disruption of AP-2alpha results in postnatal midfacial hypoplasia, vascular anomalies, and nasal cavity defects. Dev. Biol. 267, 72–92. 14975718 10.1016/j.ydbio.2003.10.033

[b44-0080031] Nottoli T. Hagopian-Donaldson S. Zhang J. Perkins A. Williams T. (1998). AP-2-null cells disrupt morphogenesis of the eye, face, and limbs in chimeric mice. Proc. Natl. Acad. Sci. USA 95, 13714–13719. 9811866 10.1073/pnas.95.23.13714 PMC24885

[b45-0080031] Parsons T. E. Kristensen E. Hornung L. Diewert V. M. Boyd S. K. German R. Z. Hallgrímsson B. (2008). Phenotypic variability and craniofacial dysmorphology: increased shape variance in a mouse model for cleft lip. J. Anat. 212, 135–143. 18093101 10.1111/j.1469-7580.2007.00845.x PMC2408978

[b46-0080031] Parsons T. E. Schmidt E. J. Boughner J. C. Jamniczky H. A. Marcucio R. S. Hallgrímsson B. (2011). Epigenetic integration of the developing brain and face. Dev. Dyn. 240, 2233–2244. 21901785 10.1002/dvdy.22729 PMC3246636

[b47-0080031] Pontoriero G. F. Deschamps P. Ashery-Padan R. Wong R. Yang Y. Zavadil J. Cvekl A. Sullivan S. Williams T. West-Mays J. A. (2008). Cell autonomous roles for AP-2alpha in lens vesicle separation and maintenance of the lens epithelial cell phenotype. Dev. Dyn. 237, 602–617. 18224708 10.1002/dvdy.21445 PMC2517426

[b48-0080031] Rahimov F. Marazita M. L. Visel A. Cooper M. E. Hitchler M. J. Rubini M. Domann F. E. Govil M. Christensen K. Bille C. (2008). Disruption of an AP-2α binding site in an IRF6 enhancer is associated with cleft lip. Nat. Genet. 40, 1341–1347. 18836445 10.1038/ng.242 PMC2691688

[b49-0080031] Rahimov F. Jugessur A. Murray J. C. (2012). Genetics of nonsyndromic orofacial clefts. Cleft Palate Craniofac. J. 49, 73–91. 21545302 10.1597/10-178 PMC3437188

[b50-0080031] Ray H. J. Niswander L. (2012). Mechanisms of tissue fusion during development. Development 139, 1701–1711. 22510983 10.1242/dev.068338 PMC3328173

[b51-0080031] Riley B. M. Mansilla M. A. Ma J. Daack-Hirsch S. Maher B. S. Raffensperger L. M. Russo E. T. Vieira A. R. Dodé C. Mohammadi M. (2007). Impaired FGF signaling contributes to cleft lip and palate. Proc. Natl. Acad. Sci. USA 104, 4512–4517. 17360555 10.1073/pnas.0607956104 PMC1810508

[b52-0080031] Schaefer S. A. Lauder G. V. (1996). Testing historical hypotheses of morphological change: biomechanical decoupling in loricarioid catfishes. Evolution 50, 1661–1675. 10.1111/j.1558-5646.1996.tb03938.x 28565701

[b53-0080031] Schmidt E. J. Parsons T. E. Jamniczky H. A. Gitelman J. Trpkov C. Boughner J. C. Logan C. C. Sensen C. W. Hallgrímsson B. (2010). Micro-computed tomography-based phenotypic approaches in embryology: procedural artifacts on assessments of embryonic craniofacial growth and development. BMC Dev. Biol. 10, 18. 20163731 10.1186/1471-213X-10-18 PMC2836989

[b54-0080031] Schorle H. Meier P. Buchert M. Jaenisch R. Mitchell P. J. (1996). Transcription factor AP-2 essential for cranial closure and craniofacial development. Nature 381, 235–238. 8622765 10.1038/381235a0

[b55-0080031] Song L. Li Y. Wang K. Wang Y. Z. Molotkov A. Gao L. Zhao T. Yamagami T. Wang Y. Gan Q. (2009). Lrp6-mediated canonical Wnt signaling is required for lip formation and fusion. Development 136, 3161–3171. 19700620 10.1242/dev.037440

[b56-0080031] Stoetzel C. Riehm S. Bennouna Greene V. Pelletier V. Vigneron J. Leheup B. Marion V. Hellé S. Danse J. M. Thibault C. (2009). Confirmation of TFAP2A gene involvement in branchio-oculo-facial syndrome (BOFS) and report of temporal bone anomalies. Am. J. Med. Genet. A 149A, 2141–2146. 19764023 10.1002/ajmg.a.33015

[b57-0080031] Subramanian A. Tamayo P. Mootha V. K. Mukherjee S. Ebert B. L. Gillette M. A. Paulovich A. Pomeroy S. L. Golub T. R. Lander E. S. (2005). Gene set enrichment analysis: a knowledge-based approach for interpreting genome-wide expression profiles. Proc. Natl. Acad. Sci. USA 102, 15545–15550. 16199517 10.1073/pnas.0506580102 PMC1239896

[b58-0080031] Tabler J. M. Barrell W. B. Szabo-Rogers H. L. Healy C. Yeung Y. Perdiguero E. G. Schulz C. Yannakoudakis B. Z. Mesbahi A. Wlodarczyk B. (2013). Fuz mutant mice reveal shared mechanisms between ciliopathies and FGF-related syndromes. Dev. Cell 25, 623–635. 23806618 10.1016/j.devcel.2013.05.021 PMC3697100

[b59-0080031] Thomason H. A. Zhou H. Kouwenhoven E. N. Dotto G.-P. Restivo G. Nguyen B.-C. Little H. Dixon M. J. van Bokhoven H. Dixon J. (2010). Cooperation between the transcription factors p63 and IRF6 is essential to prevent cleft palate in mice. J. Clin. Invest. 120, 1561–1569. 20424327 10.1172/JCI40266 PMC2860913

[b60-0080031] Trasler D. G. (1968). Pathogenesis of cleft lip and its relation to embryonic face shape in A-J and C57BL mice. Teratology 1, 33–49. 5696816 10.1002/tera.1420010106

[b61-0080031] Uslu V. V. Petretich M. Ruf S. Langenfeld K. Fonseca N. A. Marioni J. C. Spitz F. (2014). Long-range enhancers regulating Myc expression are required for normal facial morphogenesis. Nat. Genet. 46, 753–758. 24859337 10.1038/ng.2971

[b62-0080031] Wang J. Bai Y. Li H. Greene S. B. Klysik E. Yu W. Schwartz R. J. Williams T. J. Martin J. F. (2013). MicroRNA-17-92, a direct Ap-2α transcriptional target, modulates T-box factor activity in orofacial clefting. PLoS Genet. 9, e1003785. 24068957 10.1371/journal.pgen.1003785 PMC3777996

[b63-0080031] Weinberg S. M. Maher B. S. Marazita M. L. (2006). Parental craniofacial morphology in cleft lip with or without cleft palate as determined by cephalometry: a meta-analysis. Orthod. Craniofac. Res. 9, 18–30. 16420271 10.1111/j.1601-6343.2006.00339.x

[b64-0080031] Weinberg S. M. Neiswanger K. Richtsmeier J. T. Maher B. S. Mooney M. P. Siegel M. I. Marazita M. L. (2008). Three-dimensional morphometric analysis of craniofacial shape in the unaffected relatives of individuals with nonsyndromic orofacial clefts: a possible marker for genetic susceptibility. Am. J. Med. Genet. A 146A, 409–420. 18203157 10.1002/ajmg.a.32177

[b65-0080031] Weinberg S. M. Naidoo S. D. Bardi K. M. Brandon C. A. Neiswanger K. Resick J. M. Martin R. A. Marazita M. L. (2009). Face shape of unaffected parents with cleft affected offspring: combining three-dimensional surface imaging and geometric morphometrics. Orthod. Craniofac. Res. 12, 271–281. 19840279 10.1111/j.1601-6343.2009.01462.x PMC2765674

[b66-0080031] Wiley D. F. Amenta N. Alcantara D. A. Ghosh D. Kil Y. J. Delson E. Harcourt-Smith W. Rohlf F. J. St John K. Hamann B. (2005). Evolutionary morphing. Visualization 5, 431–438.

[b67-0080031] Williams T. Tjian R. (1991a). Characterization of a dimerization motif in AP-2 and its function in heterologous DNA-binding proteins. Science 251, 1067–1071. 1998122 10.1126/science.1998122

[b68-0080031] Williams T. Tjian R. (1991b). Analysis of the DNA-binding and activation properties of the human transcription factor AP-2. Genes Dev. 5, 670–682. 2010091 10.1101/gad.5.4.670

[b69-0080031] Wyszynski D. F. (2002). Cleft Lip and Palate. New York, NY: Oxford University Press.

[b70-0080031] Young N. M. Wat S. Diewert V. M. Browder L. W. Hallgrímsson B. (2007). Comparative morphometrics of embryonic facial morphogenesis: implications for cleft-lip etiology. Anat. Rec. (Hoboken) 290, 123–139. 17441205 10.1002/ar.20415

[b71-0080031] Young N. M. Hu D. Lainoff A. J. Smith F. J. Diaz R. Tucker A. S. Trainor P. A. Schneider R. A. Hallgrímsson B. Marcucio R. S. (2014). Embryonic bauplans and the developmental origins of facial diversity and constraint. Development 141, 1059–1063. 24550113 10.1242/dev.099994 PMC3929406

[b72-0080031] Zelditch M. L. Swiderski D. L. Sheets H. D. (2012). Geometric Morphometrics for Biologists, 1st edn San Diego, CA: Elsevier Academic Press.

[b73-0080031] Zhang J. Hagopian-Donaldson S. Serbedzija G. Elsemore J. Plehn-Dujowich D. McMahon A. P. Flavell R. A. Williams T. (1996). Neural tube, skeletal and body wall defects in mice lacking transcription factor AP-2. Nature 381, 238–241. 8622766 10.1038/381238a0

