# Supplementary material for: Tfap2a-dependent changes in mouse facial morphology result in clefting that can be ameliorated by a reduction in Fgf8 gene dosage
Source: Dis Model Mech. 2014 Nov 7;8(1):31–43. doi: 10.1242/dmm.017616 (PMC4283648; doi:10.1242/dmm.017616)
Supplement: Supplementary Material [file supp_8_1_31__index.html]

Tfap2a-dependent changes in mouse facial morphology result in clefting that can be ameliorated by a reduction in Fgf8 gene dosage — Supplementary Material 

# *Tfap2a*-dependent changes in mouse facial morphology result in clefting that can be ameliorated by a reduction in *Fgf8* gene dosage

## DMM017616 Supplementary Material

**Files in this Data Supplement:**

- **Supplementary Material**
