# Supplementary material for: Tfap2a-dependent changes in mouse facial morphology result in clefting that can be ameliorated by a reduction in Fgf8 gene dosage
Source: Dis Model Mech. 2014 Nov 7;8(1):31–43. doi: 10.1242/dmm.017616 (PMC4283648; doi:10.1242/dmm.017616)
Supplement: Supplementary Material [file supp_8.1.31_DMM017616.pdf]

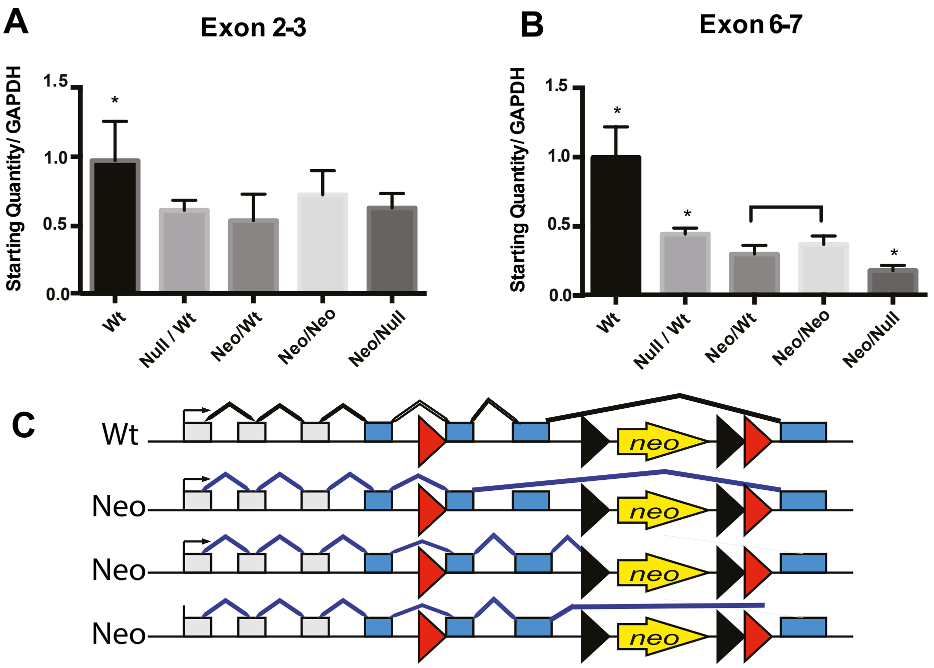

D

WT: **437 AA**

Exon 5	Exon 6	Exon 7
….GRRKAANVTLLTSLVE	GEAVHLARDFGYVCETEFPAKAVAEFLNRQHSDPNEQVARKNMLLATK	QICKEFTDLLAQDRSPLGNS…

VAR 1: Skips exon 6 **389 AA**

Exon 5	Exon 6	Exon 7
….GRRKAANVTLLTSLVE		DRSAKSSTLCWLRTDLPWGTRG PILSWSLASRVA*

VAR 2: Runs into the intron after exon 6, hits stop codon. Missing exon 7. **342 AA**

Exon 5	Exon 6	Exon 7
….GRRKAANVTLLTSLVE	GEAVHLARDFGYVCETEFPAKAVAEFLNRQHSDPNEQVARKNMLLATK*	

VAR 3: From Exon 6 splices into Neo. Alters last exon of Exon 6 from K to N and adds 10 AA from PGK promoter, missing exon 7. **352 AA**

Exon 5	Exon 6	Exon 7
….GRRKAANVTLLTSLVE	GEAVHLARDFGYVCETEFPAKAVAEFLNRQHSDPNEQVARKNMLLATNSRR AGRDKWK*	

Figure S1. Analysis of transcript levels from different regions of the *Tfap2a* locus.
qRT-PCR was performed on RNA isolated from E10.5 faces of the mouse genotypes indicated. A) Ratios of *Tfap2a* exon 2-3 levels relative to *Gapdh* and B) exon 6-7 levels relative to *Gapdh*. The ratio for the wild-type (WT) *Tfap2a* levels relative to *Gapdh* is arbitrarily set to 1. Bracket denotes samples that were not statistically different from each other, but were different from all other groups and "*" denotes P-value <0.05 from all other groups. *Tfap2a* gene organization and position of qRT-PCR assays is shown in Figure 1A. There was an ~2 fold reduction in transcripts derived from upstream exons in the various *Tfap2a* mutant genotypes compared to wild-type, and a significantly greater reduction in normal transcripts containing downstream exons, especially in the Neo/Null embryos. C) Model of the alternate splicing products derived from the Neo allele based on sequence analysis of products shown in Figure 1K. The allele can produce a wild-type transcript (top), or any of the transcript variants detected (Var1-Var 3). The splice products identified by sequencing the variant PCR products are shown on the left, and their predicted protein products are shown on the right. The blue regions in the protein product show the basic region and the helix-span-helix domains that are part of the DNA binding and dimerization domain. In all instances, the proteins produced from the aberrantly spliced products would lack a functional DNA binding and dimerization domain. D) The amino acid (AA) sequence of the wild-type protein spanning exons 5-7 is shown at the top, and the altered sequences predicted from the aberrantly spliced transcripts are shown below. An asterisk indicates a stop codon, and AA sequence shown in color illustrates protein sequence at the C-terminus unrelated to AP-2α sequence that results from aberrant splicing.

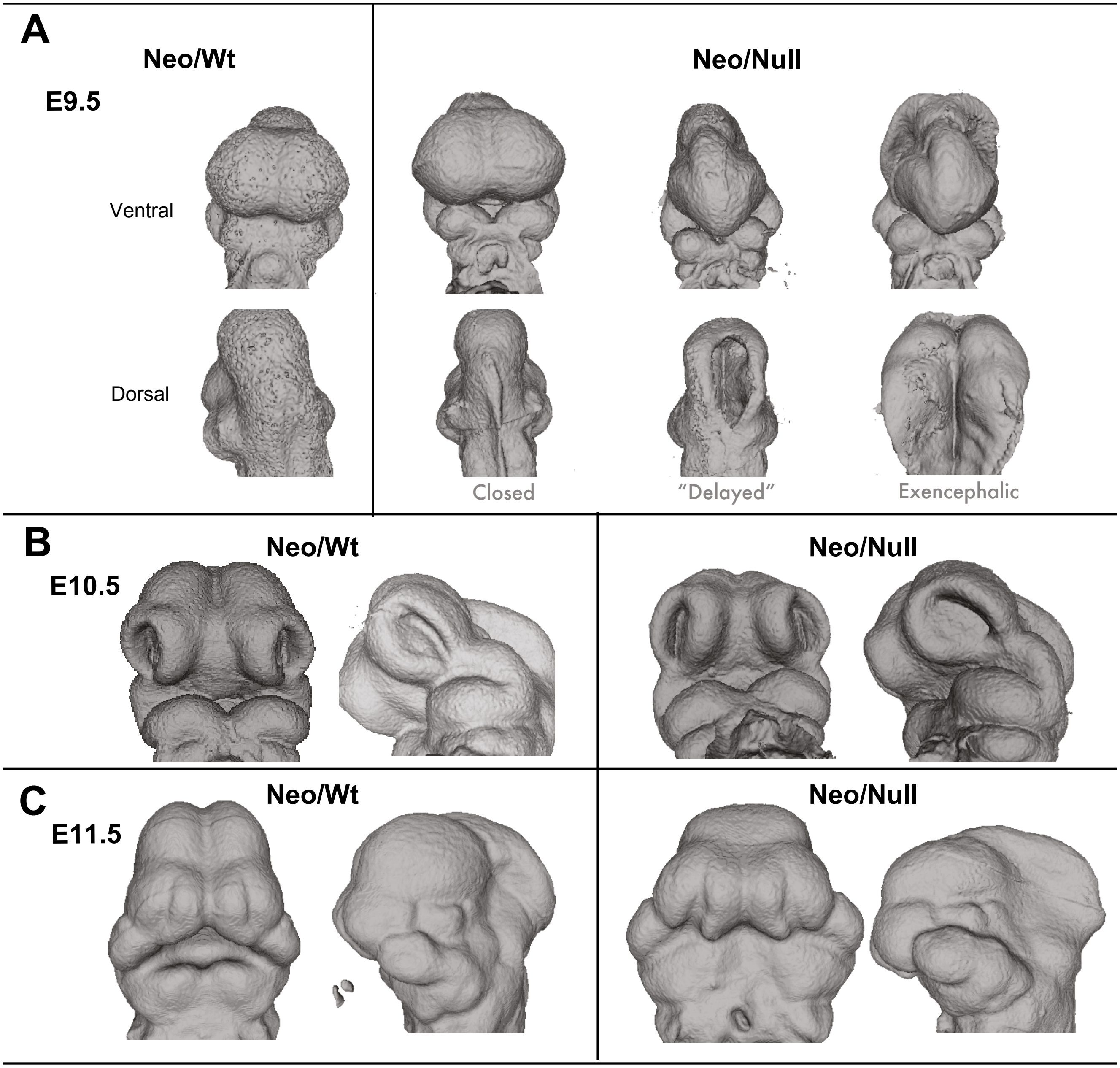

Figure S2: Sample raw scans from representative Neo/Wt and Neo/Null groups.
A) Ventral and dorsal views of the head at E9.5 showing the different classifications for neural tube closure in the Neo/Null mice. B) Ventral and lateral views of E10.5 and C) E11.5 embryos.

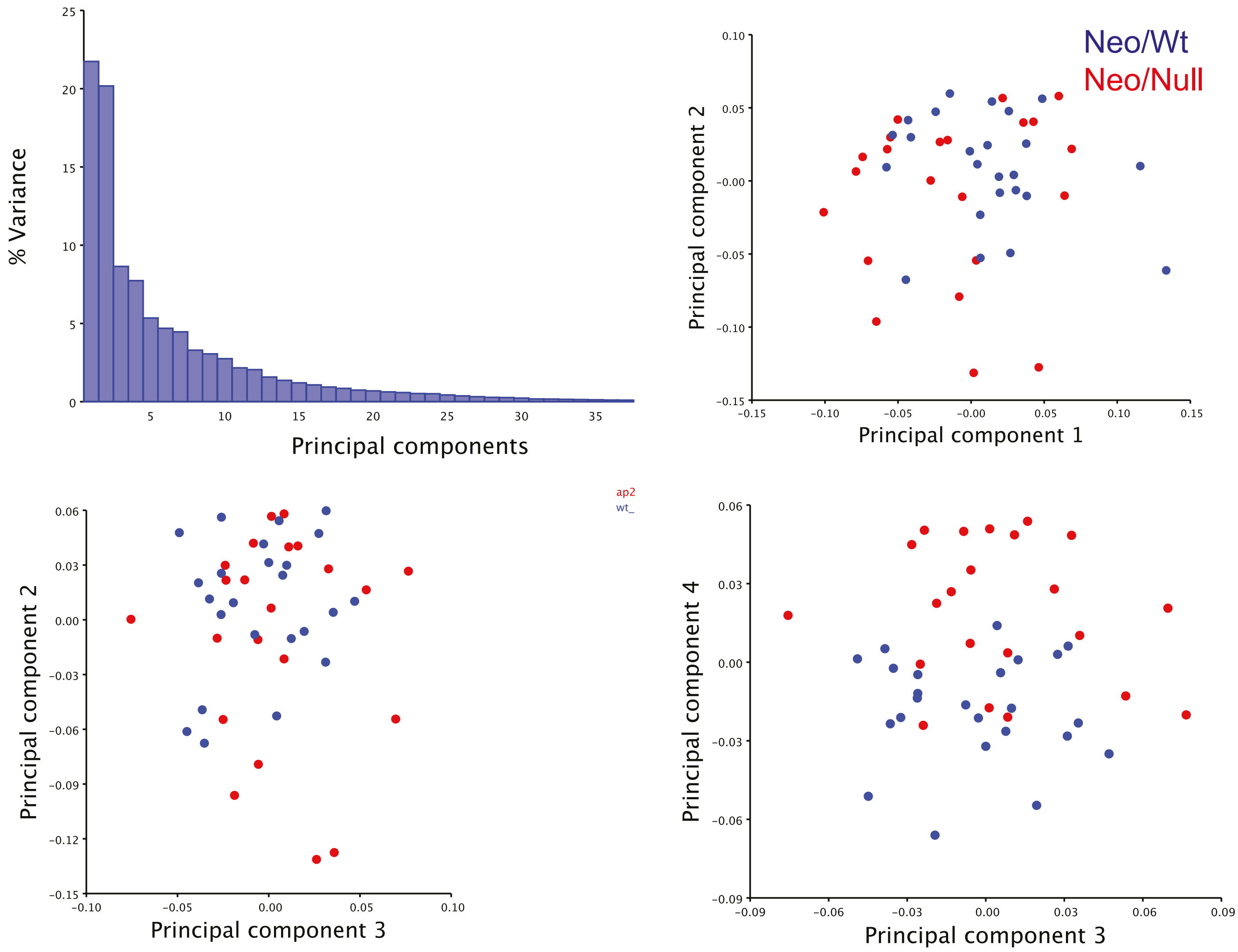

Figure S3: Principal Component Analysis from E10.5 embryos.
Neo/Null shown in red and Neo/Wt in blue. Embryos segregate by genotype along PC4, but not along PC1-3. PC4 comprises about 8% of the total variance.

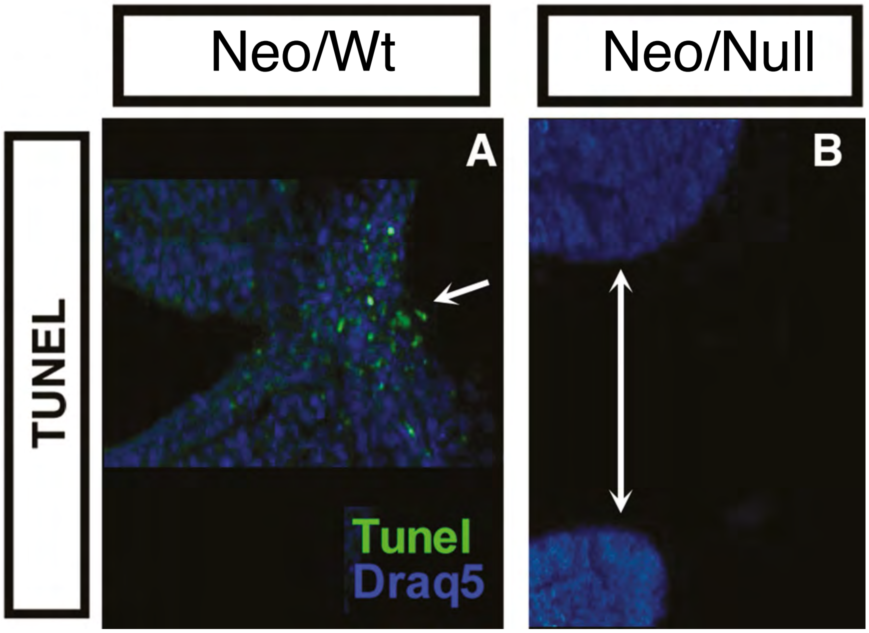

Figure S4: Apoptosis in Neo/Wt and Neo/Null nasal pit sections.
TUNEL staining (green) in the nasal pit at E10.5 for Neo/Wt (A) and Neo/Null (B) embryos. Draq5 (blue pseudo-color) is used to visualize nuclei. The white arrow indicates the fusion between the lateral and medial nasal prominences. The double headed arrow indicates the distance between the unfused prominences in Neo/Null mice.

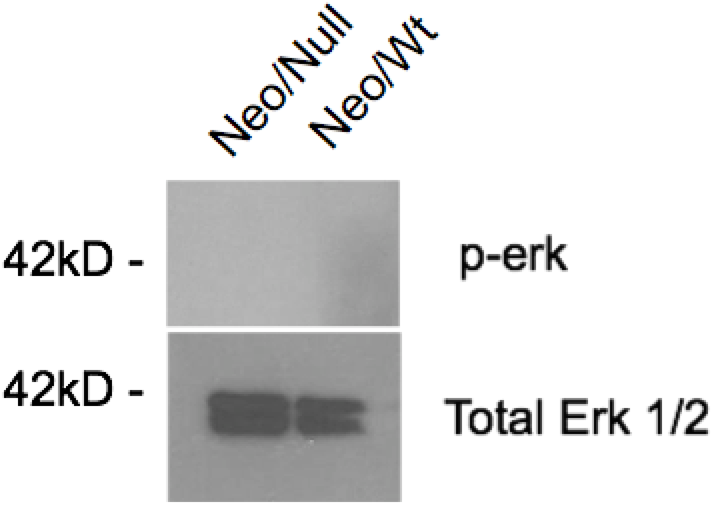

Figure S5: P-Erk and total Erk 1/2 levels in Neo/Wt and Neo/Null embryos.
Phosphatase inhibitor treated lysates from E10.5 dissected faces were probed for phospho-Erk and total Erk1 and Erk2. Microarray data analysis coupled with Western blotting indicated that there are no significant changes in *Mapk1* or Erk levels between Neo/Wt or Neo/Null mice. With respect to phospho-Erk, we could not detect these modified protein isoforms above background using this assay in either mouse sample and so it was not possible to determine if this showed a significant change in the Neo/Null sample from the Neo/Wt.

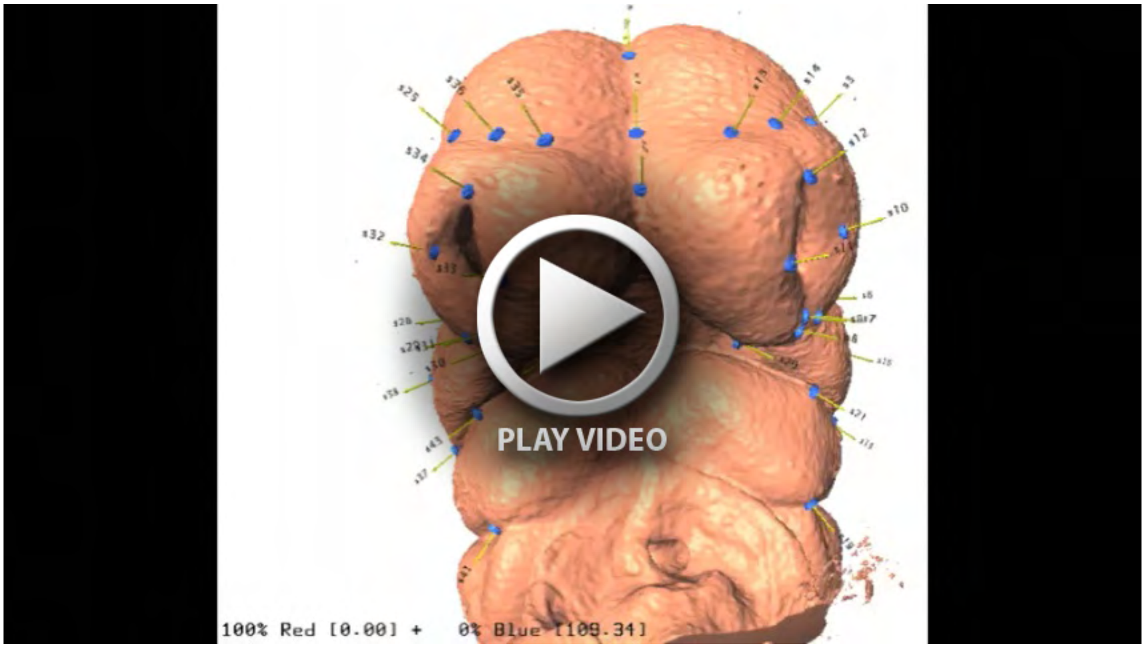

Movie S1: Mathematical timelapse of facial development in Neo/Wt embryos.
Movie shows timelapse between E10.5-E11.5 for Neo/Wt end of PC4 (E10.5) to the Neo/Wt end of PC1 (E11.5).

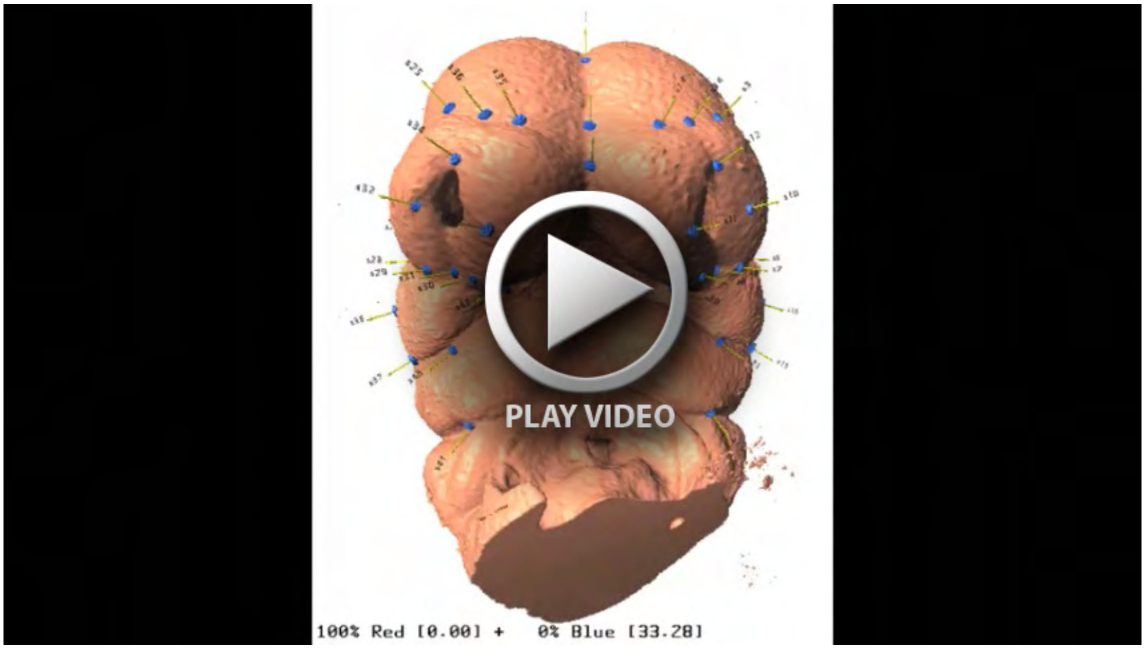

Movie S2: Mathematical timelapse of facial development in Neo/Null embryos.
Movie shows timelapse between E10.5-E11.5 for Neo/Null end of PC4 (E10.5) to the Neo/Null end of PC1 (E11.5).

Supplemental Table 1:

List of landmarks for E9.5 embryos.
Numbers in parentheses represent the right side of the embryo.

1) Junction between forebrain and midbrain along the center line
2) Maximum of curvature along center line between pt 1 and most rostral point.
3) Most ventral point along midline of the frontonasal prominence (FNP)
4) Maximum of curvature along the central line on ventral side of the FNP.
5) (13) Maximum of curvature along the lateral edge of the FNP as taken from a rostral view
6) (14) Midpoint of the lateral nasal prominence (LNP) from a lateral view
7) (15) Midpoint between the LNP and the maxilla
8) (16) Maximum of the maxilla from a lateral view
9) (17) Midpoint between the maxilla and the mandibular prominence
10) (18) Maximum of curvature of the mandibular prominence from a lateral view
11) (19) Between the first and second arch at the base of the maxillary prominence
12) (20) Between the first and second arch at the base of the mandibular prominence

Supplemental Table 2:

P-values from permutation tests (10000 permutation rounds) for Procrustes distances between groups from CVA tests (Figure 2A). Note that all “between group differences” reach statistical significance at a P value of <0.05.

E9.5	Neo/Null closed	Neo/Null exencephaly	Neo/Null open
Neo/Null exencephaly	0.0001		
Neo/Null open	0.0190	0.0014	
Neo/Wt closed	0.0074	0.0001	0.0091

E11.5	Neo/Null closed	Neo/Null exencephaly
Neo/Null exencephaly	0.0123	
Neo/Wt closed	0.0213	0.0001

Supplemental Table 3:

Results of microarray analysis of gene expression in E10.5 nasal prominences performed in triplicate on independent sample pools. Attached spreadsheet shows list of genes in the nasal process with a present call in both Neo/Null (nnb) and Neo/Wt (wnb) groups, showing a >1.25 fold difference, and a P-value of <0.05.

Download Table S3

Supplemental Table 4:

Results of microarray analysis of gene expression in E10.5 maxillary prominence performed in triplicate on independent sample pools. Attached spreadsheet shows list of genes in the maxilla with a present call in both Neo/Null (nxb) and Neo/Wt (wxb) groups, showing a >1.25 fold difference, and a P-value of <0.05.

Download Table S4

Supplemental Table 5:

Parametric P-values from angular comparisons of 2-block PLS scores. Block 1 was the nasal prominence landmarks and block 2 was the maxillary prominence landmarks. The MorphoJ compare vector test was used to examine differences between groups. Statistically significant values are shown in blue.

PLS1				
	C57	Neo/Null;Fgf8het	Neo/Null;Fgf8wt	Neo/Wt; Fgf8het
Neo/Null;Fgf8het	0.035			
Neo/Null;Fgf8wt	0.00064	0.00518		
Neo/Wt;Fgf8het	0.18634	0.00004	0.00584	
Neo/Wt;Fgf8wt	<0.00001	0.00246	0.00013	0.15957
PLS2				
	C57	Neo/Null;Fgf8het	Neo/Null;Fgf8wt	Neo/Wt; Fgf8het
Neo/Null;Fgf8het	0.0041			
Neo/Null;Fgf8wt	0.96972	0.00329		
Neo/Wt;Fgf8het	0.16566	0.80168	0.28387	
Neo/Wt;Fgf8wt	0.41998	0.00002	0.05811	0.00065
